# 3A Cyanide‐Bridged Ru–Fe Supramolecular Photocatalyst for Efficient CO_2_ Reduction

**DOI:** 10.1002/cssc.70969

**Published:** 2026-08-13

**Authors:** Francesca Mancini, Alberto Bianco, Albert Ruggi, Giacomo Bergamini

**Affiliations:** ^1^ Department of Chemistry “Giacomo Ciamician” University of Bologna Bologna Italy; ^2^ Department of Chemistry University of Fribourg Fribourg Switzerland

**Keywords:** CO_2_ reduction, cyanide bridging, electron transfer, iron catalysis, supramolecular photocatalysis

## Abstract

Supramolecular control over electron transfer is a key challenge in artificial photosynthesis. Here, we report a cyano‐bridged Ru–Fe supramolecular photocatalyst that self‐assembles in solution through spontaneous coordination. This preorganized architecture enables ultrafast excited‐state electron transfer from Ru to Fe via a static oxidative quenching pathway, as revealed by pump‐probe transient absorption spectroscopy showing sub‐picosecond formation of the reduced Fe species, in contrast to the reductive quenching mechanisms commonly observed in related systems. Remarkably, reversible coordination of CO_2_ to the supramolecular adduct can be directly monitored by electronic absorption spectroscopy, providing rare experimental access to substrate binding within a molecular photocatalytic system. Under visible‐light irradiation, the system achieves highly efficient CO_2_‐to‐CO conversion (AQY 18%), with CO selectivity exceeding 95%, suggesting an intrinsic preference of the supramolecular photocatalyst for CO formation. These findings establish cyano bridging as a powerful design principle for constructing supramolecular photocatalysts, enabling both mechanistic transparency and enhanced performance. More broadly, this strategy defines a modular platform that can be extended to a wide range of catalysts and photocatalytic transformations.

## Graphical abstract

A cyanide‐bridged Ru–Fe supramolecular photocatalyst self‐assembles in solution, enabling ultrafast electron transfer via an oxidative quenching pathway. The system allows direct spectroscopic detection of reversible CO_2_ binding and achieves highly selective CO_2_‐to‐CO conversion, revealing a modular strategy to control electron transfer and reactivity in molecular photocatalysis.

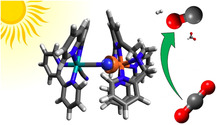

## 1 Introduction

The growing need to mitigate CO_2_ emissions while developing sustainable energy vectors has stimulated intense efforts toward artificial photosynthesis [1]. In this context, photocatalytic CO_2_ reduction offers an attractive strategy to convert solar energy into fuels and value‐added chemicals [2, 3]. However, the activation of CO_2_ remains challenging, since its first one‐electron reduction is highly endergonic (*E*° ≈ −2.15 V vs. SCE in aqueous media) [4]. The introduction of proton sources can lower the thermodynamic barrier; however, it often promotes the competing hydrogen evolution reaction (HER) with a consequent loss of product selectivity [2, 5]. Efficient molecular systems must therefore couple fast multi‐electron, multi‐proton management with high product selectivity.

Molecular catalysis has made significant progress toward this goal, particularly through the design of redox‐active ligands capable of stabilizing low‐valent metal species [6, 7] or storing reducing equivalents within conjugated frameworks [8, 9]. In parallel, first‐row transition metals have gained increasing attention as sustainable catalytic centers, with iron complexes emerging as promising candidates for selective CO_2_‐to‐CO conversion [10, 12].

Photocatalytic CO_2_ reduction is most commonly carried out using dynamic systems, in which the photosensitizer and the catalyst diffuse freely in solution [2, 7, 13, 14]. In contrast, supramolecular photocatalysts, where the light‐absorbing and the catalytic units are preassembled through covalent or noncovalent interactions, often display distinct advantages, including accelerated electron transfer, improved photosensitizer stability, and enhanced turnover frequencies. Seminal examples of covalently linked dyads have been widely reported [15, 18], whereas only a limited number of systems exploit noncovalent assembly to achieve improved CO_2_‐to‐CO efficiency, typically via hydrogen bonding [19] or π–π interactions [20, 21].

**FIGURE 1:**
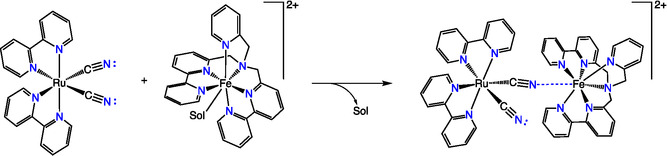
Cyano‐bridged supramolecular interaction between the photosensitizer [Ru(bpy)_2_(CN)_2_] (**Ru**) and the catalyst [Fe(DBPy‐PyA)]^2+^ (**Fe**).

Here, we propose a distinct supramolecular strategy based on cyano‐bridge coordination. The photosensitizer [Ru(bpy)_2_(CN)_2_] (**Ru**), (bpy = 2,2′‐bipyridine), is well known for its tunable photophysics and for the ability of its cyanide ligands to bind additional metal centers [22, 24]. Although **Ru**‐based cyano‐bridged adducts have been extensively investigated [25, 26], their potential in photocatalysis has remained largely unexplored. The catalyst [Fe(DBPyPyA)](BF_4_)_2_ (**Fe**), where DBPyPyA = 1‐([2,2′‐bipyridin]‐6‐yl)‐N‐([2,2′‐bipyridin]‐6‐ylmethyl)‐N(pyridin‐2‐ylmethyl) methanamine, has recently been shown to be an efficient and selective CO_2_‐to‐CO catalyst in dynamic photocatalytic systems [11, 12]. In this complex, Fe^2+^ adopts an unusual heptacoordinate geometry, with six coordination sites occupied by the hexadentate DBPyPyA ligand, and a seventh one accessible to an additional ligand such as solvent molecule. This property can be fruitfully exploited for the formation of supramolecular adducts [4, 11, 12]. In this work, we demonstrate that **Ru** and **Fe** spontaneously assemble into a supramolecular adduct through coordination of the cyanide ligand, without any additional synthetic effort (Figure 1). We then assess how this preorganization influences the photocatalytic reduction of CO_2_ in the presence of a sacrificial donor, highlighting the advantages conferred by this supramolecular approach.

## 2 Results and Discussion

### 2.1 Supramolecular Interaction Study

The photophysical properties of the photosensitizer **Ru** and the catalyst **Fe** were examined to provide a basis for interpreting their supramolecular interaction and subsequent photocatalytic behavior. In acetonitrile, **Ru** exhibits the characteristic metal‐to‐ligand charge transfer absorption band (^1^MLCT) at 496 nm together with intense ligand‐centered transitions in the UV region (Figure S1). Excitation of **Ru** yields a broad ^3^MLCT emission band centered at 695 nm with a lifetime of 65 ns in aerated conditions. The presence of two cyanide ligands not only modulates the MLCT energy but also confers high sensitivity to Lewis‐acid interactions at the CN site [25, 27, 28]. In agreement with literature observations, **Fe** displays weak d–d transitions in the visible region, with a band centered at 476 nm (*ε* ≈ 800 M^−1^·cm^−1^), and dominant ligand‐based absorptions at higher energies (Figure S2) [4]. Moreover, no emissive behavior is observed under any of the conditions examined, including in deoxygenated media and at low temperature.

**FIGURE 2:**
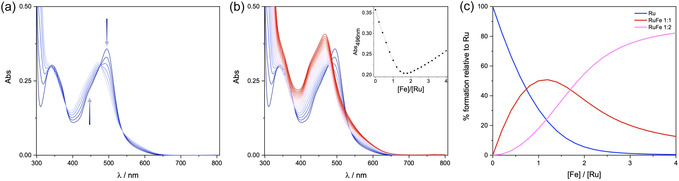
**Ru**–**Fe** association process investigation through absorption spectroscopy. (a) Initial stage of the absorption titration of a 2 mL **Ru** solution (36 μM) with **Fe** (2.8 mM) in acetonitrile, showing the first equivalent of **Fe** added. Over this first equivalent, a well‐defined isosbestic point is maintained. (b) Full absorption titration of the same **Ru** solution with up to four equivalents of **Fe** (2.8 mM) in acetonitrile. Inset: fitting of the absorbance evolution at 496 nm as a function of added **Fe** equivalents. (c) Speciation diagram showing the relative distribution of **Ru**‐containing species (expressed as a percentage of total **Ru**) as a function of **Fe** equivalents added.

The interaction between **Ru** and **Fe** was first investigated by UV–vis absorption titration, performed by gradually adding a 2.8 mM **Fe** solution to a 36 μM **Ru** solution in acetonitrile, up to a total of four equivalents. Upon **Fe** addition, the ^1^MLCT band of **Ru** at 496 nm progressively decreases, while new absorption features appear at 466 and 570 nm (Figure 2a,b). These spectral changes are accompanied by the formation of a clean isosbestic point at 475 nm, indicating an interconversion between two distinct species (Figure 2a). The isosbestic point remains stable up to 1 equivalent of **Fe** and is no longer maintained at higher **Fe** loadings due to the growth of the absorption feature at 570 nm (Figure 2b).

The observed blueshift of the ^1^MLCT band of **Ru** is attributed to coordination of **Fe** to the **Ru**‐bound cyano ligands, with the **Fe** center acting as a Lewis acid. Similar effects have been reported for cyano‐bridged **Ru** adducts with Ag^+^ and Pt^2+^ salts [25, 27], as well as upon protonation [28, 29], underscoring the pronounced sensitivity of Ru‐cyanide ^1^MLCT transitions to interactions at the CN donor site. The association is best described by the stepwise formation of 1:1 and 1:2 **Ru–Fe** adducts, in line with the presence of two CN ligands in **Ru** [25]. Speciation analysis (Figure 2c) confirms this model: after the addition of 4 equivalents of **Fe**, no free **Ru** remains, with 13% of the 1:1 complex and 87% of the 1:2 species present. The equilibrium constants obtained from the global fitting [30], *K*_ass1_ = 4 × 10^5^ M^–1^ and *K*_ass2_ = 1 × 10^5^ M^–1^, have comparable magnitudes, indicating that formation of the first adduct does not hinder coordination of a second **Fe** center. The involvement of kinetic barriers in the association process can be ruled out, as no additional spectral changes were observed even when equilibration times of up to 30 min were allowed between successive additions of the iron complex.

**FIGURE 3:**
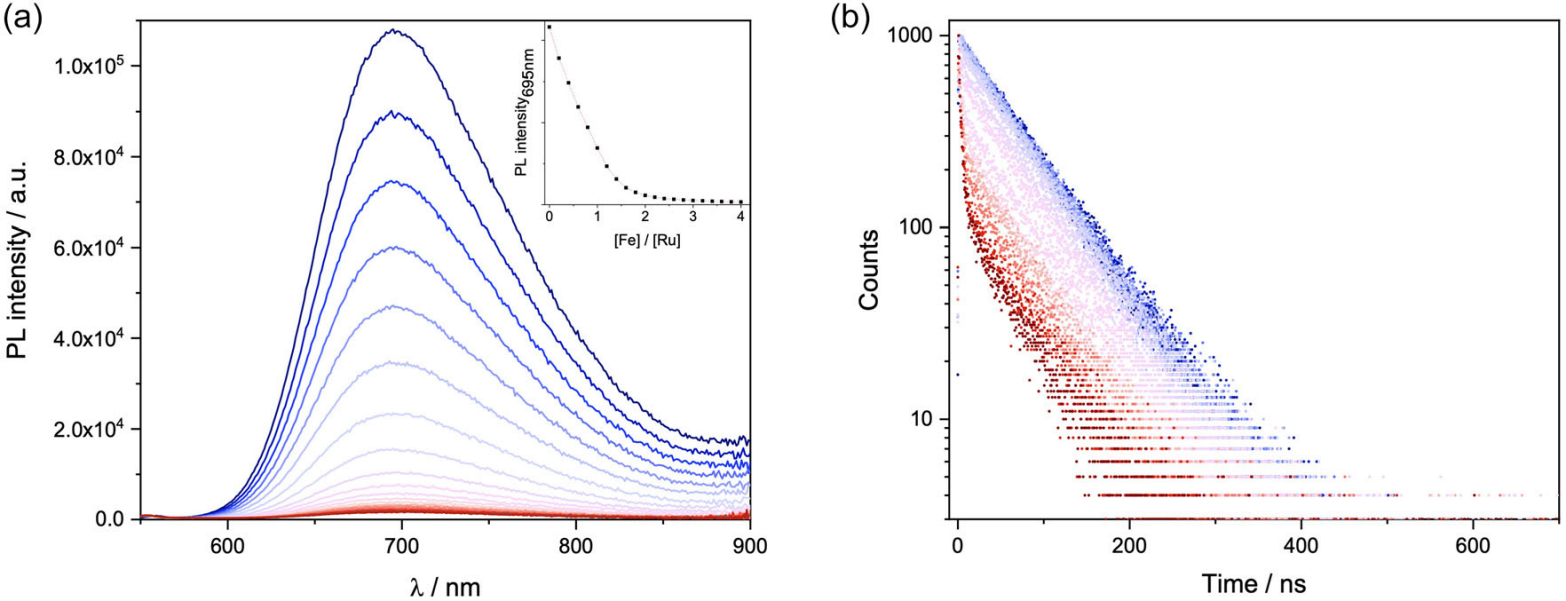
**Ru**–**Fe** association process investigation through (a) steady‐state (*λ*_exc_ = 475 nm, inset: fitting of the emission intensity evolution at 695 nm as a function of added **Fe** equivalents) and (b) time‐resolved emission spectroscopy on **Ru** (36 μM) with **Fe** (2.8 mM) as titrant in acetonitrile.

Following the same titration with both steady‐state and time‐resolved emission techniques, we observed that **Ru** ^3^MLCT emission is statically quenched upon **Fe** addition. Specifically, the steady‐state measurement (Figure 3a) shows that emission maximum at 695 nm decreases monotonically and levels off at a plateau, consistent with complete formation of the supramolecular adducts. Time‐resolved measurement (Figure 3b) shows that the **Ru** excited‐state lifetime remains unchanged (*τ* = 65 ns) throughout the entire titration. This behavior rules out dynamic quenching and unambiguously indicates that emission quenching originates from static interactions, namely the formation of ground‐state **Ru–Fe** supramolecular adducts.

To ensure that the observed changes arise exclusively from quenching phenomena, all photoluminescence spectra were acquired by exciting the sample at 475 nm, corresponding to the isoabsorbing point of the system, thereby maintaining a constant photon absorption throughout the titration.

The formation of **Ru–Fe** adducts was further corroborated by mass‐spectrometric and IR spectroscopic techniques. In particular, high‐resolution electrospray ionization mass spectrometry (HR‐ESI) provided direct evidence for **Ru–Fe** supramolecular species (*m*/*z* = 483.09449), in excellent agreement with the calculated mass of [**Ru–Fe**]^2+^ (see Figure S3). Attenuated total reflection‐Fourier transform infrared (ATR‐FTIR) spectra revealed diagnostic shifts of the CN stretching bands. Upon adduct formation, the primary maximum at 2054 cm^–1^ and the secondary shoulder at 2065 cm^–1^ shift to 2056 and 2070 cm^–1^, respectively (Figure S4), as expected for coordination of a metal with the CN ligand [25].

**FIGURE 4:**
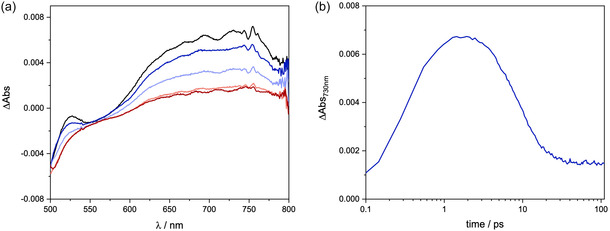
(a) Decay spectra acquired at 1.6, 5.6, 10.6, 22.2, and 30.5 ps, in the order from black to red curve, (b) kinetic trace at 730 nm of reduced Fe^−^ species from ultrafast transient absorption spectroscopy measurement.

Based on the excited‐state oxidation potential of **Ru**, calculated within the approximation of the thermally equilibrated excited state (*E* = –1.68 V vs. SCE in acetonitrile) [26, 31] and the **Fe** reduction potential (*E* = –1.49 V vs. SCE in acetonitrile) [4], an oxidative quenching pathway for the photosensitizer can be proposed. This hypothesis was supported by ultrafast transient absorption spectroscopy, which revealed the formation of the reduced **Fe** species from the excited state of **Ru**. The appearance of the **Fe^−^** signal, consistent with previously reported spectral fingerprints [4, 11], occurs on a sub‐picosecond timescale, indicating an ultrafast electron‐transfer process from the **Ru** excited‐state to **Fe** within the supramolecular adduct (Figure 4a). The reduced **Fe** ^**–**^ intermediate subsequently decays with a lifetime of approximately 9 ps (Figure 4b), consistent with rapid charge recombination or further electron‐transfer steps inside the assembly (Figure S6). These observations provide direct spectroscopic evidence for oxidative quenching in the **Ru–Fe** supramolecular system [11]. Interestingly, this behavior contrasts with most supramolecular photocatalysts reported to date, which predominantly operate through reductive quenching pathways [15, 17, 19, 20].

### 2.2 Light‐Driven CO_2_ Reduction Investigation

The light‐driven CO_2_ reduction capability of the dyad **Ru–Fe** was investigated in the presence of the sacrificial reagents 3‐dimethyl‐2‐phenylbenzimidazoline (BIH) and triethanolamine (TEOA). BIH operates as a two‐electron, one‐proton (hydride) donor, as detailed in Section S5, Supporting Information. Following photoinduced electron transfer from **Ru** to **Fe**, BIH reduces the oxidized photosensitizer (PS^+^), regenerating the ground‐state Ru and producing BIH^•+^ [32]. The latter is strongly acidic, and its rapid deprotonation by TEOA suppresses back‐electron transfer, enabling irreversible regeneration of the photosensitizer [33].

**FIGURE 5:**
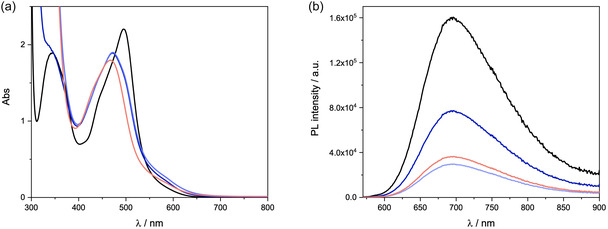
(a) Absorption and (b) emission spectra (*λ*_exc_ = 475 nm) of the photocatalytic system upon consequential addition of each component. Ru 232 μM (black spectrum), Fe 185 μM addition (dark blue spectrum), BIH 0.1 M addition (light blue spectrum), TEOA 0.3 M addition (pink spectrum).

To probe the sequence of elementary steps within the photocatalytic cycle, the photophysical response of the system was monitored upon stepwise addition of each component (Figure 5).

Upon addition of **Fe** to **Ru**, the ^1^MLCT absorption band undergoes the hypsochromic shift described above, accompanied by the appearance of a shoulder at ≈570 nm. In parallel, a pronounced quenching of the Ru ^3^MLCT emission is observed, consistent with the formation of ground‐state **Ru–Fe** supramolecular adducts and the associated static quenching pathway.

Subsequent addition of BIH produces negligible changes in the absorption spectrum, indicating the absence of significant ground‐state interactions. In contrast, emission measurements reveal further quenching, accompanied by a decrease in the excited‐state lifetime from 65 to 32 ns (Figure S7). This behavior indicates the onset of dynamic quenching, attributable to diffusional interaction between BIH and the fraction of unassociated **Ru** species in solution. Thus, BIH operates in parallel with the static quenching pathway imposed by the **Ru–Fe** assembly. Upon addition of TEOA, only minor changes are observed in the absorption spectrum, while a slight increase in emission intensity is detected. The excited‐state lifetime remains essentially unchanged (Figure S9), indicating that TEOA does not participate in excited‐state quenching. The observed spectral variations are therefore attributed to polarity effects rather than to direct interaction with *Ru. This interpretation is supported by control experiments (Figure S10), which confirm that TEOA does not quench the **Ru** ^3^MLCT excited state.

Overall, these results support a photocatalytic mechanism in which photoexcitation of **Ru** is followed by intramolecular electron transfer to the **Fe** center within the supramolecular assembly, while BIH and TEOA ensure efficient regeneration of the photosensitizer.

**FIGURE 6:**
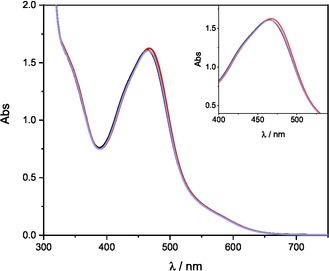
Reversible absorption spectral changes of the preassembled **Ru–Fe** supramolecular complex upon CO_2_ binding in the presence of TEOA; **Ru–Fe** and 0.3 M TEOA (black curve), **Ru–Fe** and 0.3 M TEOA after CO_2_ bubbling (red curve), **Ru–Fe** + TEOA after argon purging (blue curve).

Having elucidated the initial photophysical processes, we next introduced CO_2_ as the reaction substrate to probe potential interactions with the supramolecular assembly. Upon bubbling CO_2_ into a solution of the **Ru–Fe** adduct in the presence of TEOA, a slight redshift accompanied by an increase in intensity of the **Ru** ^1^MLCT band is observed (Figure 6). These spectral changes are consistent with the coordination of CO_2_ at the catalytic Fe center. Importantly, subsequent purging of the solution with argon restores the original absorption profile, demonstrating the reversibility of the interaction and providing direct spectroscopic evidence for CO_2_ association with the **Ru–Fe** assembly under photocatalytically relevant conditions [34].

Such direct observation of substrate coordination remains rare in molecular photocatalytic systems. Notably, this sensitivity arises from the supramolecular organization of the **Ru–Fe** dyad, where strong electronic coupling enables coordination events at the catalytic site to be transduced into measurable changes in the Ru‐centered MLCT transition [29]. Additional spectroscopic and electrochemical evidence for CO_2_ coordination to the **Ru–Fe** complex is provided in the Supporting Information (Figures S11 and S12).

Having established the key photophysical and mechanistic features of the system, we then assessed its photocatalytic performance under optimized conditions.

The reaction was carried out in 3 mL acetonitrile solution containing **Ru** (232 μM), **Fe** (185 μM, corresponding to 0.8 equivalents relative to **Ru**), BIH (0.1 M), and TEOA (0.3 M). The relatively high **Ru** concentration ensures complete absorption (Abs > 2) of the 475 nm excitation light throughout the irradiated volume.

**FIGURE 7:**
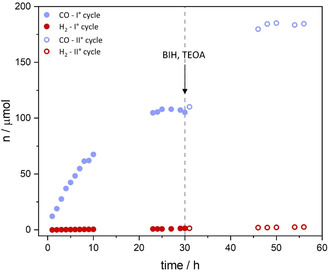
First and second cycle kinetics of CO and H_2_ evolution over 56 h of irradiation under 475 nm LED. The gray dashed line at 30 h indicates the BIH and TEOA addition and the start of the second cycle.

The reaction mixture was irradiated for 30 h with a 475 nm LED. The kinetic traces display an initial linear regime followed by a plateau after approximately 20 h (Figure 7). After 30 h of irradiation, 107 μmol of CO, 2.8 μmol of HCOO^−^, and 1.3 μmol of H_2_ were produced, corresponding to a CO selectivity of >95%. The apparent quantum yield (AQY, calculated as described in Section 8 of Supporting Information) is 18%, and the turnover number for CO formation reaches TON_CO_ = 192. Notably, the amounts of formate and H_2_ detected under photocatalytic conditions are comparable to those observed in control experiments (see below), suggesting that their formation largely arises from background processes rather than from the **Ru–Fe** photocatalytic cycle. Accordingly, the intrinsic selectivity of the supramolecular system toward CO formation is likely even higher than the observed value, as the small amounts of H_2_ and HCOO^–^ detected are comparable to those generated through background processes in the control experiments.

The initial amount of BIH (300 μmol) theoretically supports the formation of up to 150 μmol of reduced products, given that each CO_2_ reduction or H_2_ evolution event requires two electrons and two protons, corresponding to two equivalents of BIH. Under the optimized conditions, the cumulative yield of reduction products (CO + HCOO^−^ + H_2_) reaches 111 μmol, corresponding to 74% of this theoretical maximum.

This result highlights the efficient utilization of the sacrificial donor. In this system, BIH acts as the limiting reagent, serving as the sole source of both electrons and protons; consequently, the plateau observed in the kinetic profile is primarily attributed to its depletion rather than to catalyst deactivation. This interpretation is further supported by postirradiation spectroscopic analysis, which confirms the structural integrity of the catalytic system (Figure S14).

To further evaluate the robustness of the **Ru–Fe** supramolecular assembly and confirm that the sacrificial donor is the primary factor limiting CO_2_ reduction, a second irradiation cycle was performed. Following the initial 30 h of reaction, the system was supplemented with fresh BIH (0.1 M) and TEOA (0.3 M). After re‐equilibrating the solution with CO_2_, irradiation was resumed for an additional 26 h. Furthermore, Figure 7 shows also the kinetic profile for the second catalytic cycle: over a total of 56 h of irradiation, 185 μmol of CO, 5.6 μmol of HCOO^−^, and 2.5 μmol of H_2_ were produced. CO selectivity remains constant at >95%, and the cumulative TON_CO_ reaches 362. These results demonstrate that the supramolecular assembly retains catalytic activity over extended operation, highlighting the difference between catalyst deactivation and the exhaustion of sacrificial reagents.

Absorption spectra recorded after 56 h of irradiation (Figure S14) confirm the overall photostability of the supramolecular system, with the Ru‐based ^1^MLCT band largely preserved and only a slight decrease in intensity. This minor attenuation suggests limited photosensitizer degradation under prolonged irradiation. A modest decrease in emission intensity is also observed, correlating with the reduced CO production during the second cycle (≈70% of the initial 30‐h output).

Such behavior likely reflects partial loss of photosensitizer efficiency, which may be exacerbated by the progressive depletion of BIH, reducing the effectiveness of excited‐state regeneration at later stages of the reaction. Overall, these results indicate that the diminished activity upon recycling arises primarily from photosensitizer fatigue rather than catalyst deactivation.

To validate the photocatalytic cycle and clarify the role of each component, a comprehensive series of control experiments was performed (see Section S10, Supporting Information). Specifically, the systematic omission of individual components (**Ru**, **Fe** catalyst, BIH, or TEOA) yielded no detectable CO formation. These results, summarized in Figure S17, confirm that CO evolution is strictly dependent on the integrity of the complete multicomponent system under a CO_2_ atmosphere. In particular, irradiation of the reaction mixture under argon instead of CO_2_ resulted in no CO production, unequivocally demonstrating that CO originates from CO_2_ reduction. A small amount of formate (5.1 μmol) was detected under these conditions and is attributed to minor photoinduced degradation pathways. This interpretation is supported by the significant decrease in Ru absorption and emission observed after irradiation in the absence of CO_2_ (Figure S15), indicating reduced system stability without substrate coordination. In the absence of BIH, neither CO_2_ reduction products nor H_2_ evolution were observed, confirming its essential role as sacrificial electron and proton donor. Omitting TEOA completely suppresses CO formation and yields 16 μmol of formate together with only trace H_2_. These results highlight the critical function of TEOA in enabling efficient BIH oxidation through deprotonation of BIH^•+^ radical cation. Formate and minor H_2_ evolution in its absence suggest alternative proton‐transfer pathways may operate under these conditions, although their mechanistic details remain to be clarified.

Photocatalytic experiments performed without **Fe** yielded neither CO nor formate and only negligible H_2_ evolution (0.09 μmol), confirming that the iron complex is required for productive CO_2_ reduction. Replacement of **Fe** with Fe(BF_4_)_2_·6H_2_O resulted in poor photostability (Figure S16) and strongly diminished activity, with no detectable CO_2_ reduction products and only ≈15 μmol of H_2_ after 30 h. These findings demonstrate that the DBPy‐PyA ligand scaffold is essential for maintaining catalytic integrity and facilitating efficient electron transfer. The formation of catalytically active nanoparticles was ruled out by performing dynamic light scattering (DLS) on the irradiated solutions (Figure S18), which confirmed the homogeneous nature of the system.

**FIGURE 8:**
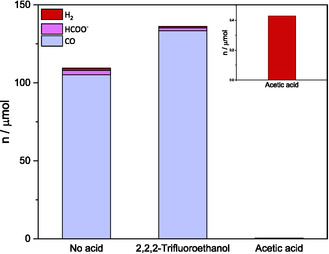
CO_2_ reduction products and H_2_ formation after 30 h of irradiation in the presence of different proton donors of increasing acidic strength: BIH (0.1 M), trifluoroethanol (0.375 M), and acetic acid (0.375 M). Inset: zoom on the H_2_ production when acetic acid is the proton source.

To probe the influence of proton‐donor acidity on catalytic performance, two acids with different *pK*_a_ in acetonitrile were examined: trifluoroethanol (TFE, *pK*_a_ = 35.4) and acetic acid (*pK*_a_ = 23.5) [35]. This comparison allows evaluation of how proton strength affects product distribution and catalytic efficiency (Figure 8).

Consistent with previous reports [12, 36, 37], addition of 0.375 M TFE enhances CO production from 107 to 133 μmol and increases selectivity from 96% to 98%, while maintaining minimal formate (1.8 μmol) and H_2_ (0.9 μmol) formation. In contrast, the addition of acetic acid completely suppresses CO_2_ reduction, with neither CO nor formate detected. Notably, no significant increase in H_2_ evolution is observed, indicating that strongly acidic conditions inhibit the overall catalytic cycle rather than redirecting it toward hydrogen evolution. This behavior is consistent with the intrinsically low HER activity of the **Fe** catalyst [11].

## 3 Conclusion

This work presents a supramolecular photocatalyst formed through the spontaneous cyano‐bridged assembly of [Ru(bpy)_2_(CN)_2_] (**Ru**) and the heptacoordinated iron complex [Fe(DBPyPyA)](BF_4_)_2_ (**Fe**). In solution, the two components form well‐defined 1:1 and 1:2 adducts that enable efficient oxidative quenching of the **Ru** excited state and rapid electron transfer to the **Fe** center. Direct spectroscopic observation of both ultrafast **Fe** reduction and reversible CO_2_ coordination to the supramolecular adduct highlights the unique mechanistic insights afforded by this preorganized architecture.

Under optimized conditions, the **Ru–Fe** system achieves highly selective CO formation (>95%), an apparent quantum yield of 18%, and a cumulative TON of 362 over two irradiation cycles, demonstrating good stability and recyclability. Control experiments confirm the essential roles of BIH, TEOA, and the **Fe** ligand framework, while proton‐donor studies underscore the importance of controlled acidity in maximizing CO_2_‐to‐CO conversion.

Overall, these findings establish cyano coordination as a simple and versatile strategy for preassembling photosensitizer–catalyst pairs, providing enhanced mechanistic insight and improved performance in molecular CO_2_ photoreduction. Moreover, this approach defines a supramolecular platform with intrinsic modularity, enabling straightforward adaptation to a broad range of molecular catalysts and photocatalytic transformations beyond CO_2_ reduction.

## Funding

This work was supported by European Union—NextGenerationEU (iENTRANCE@ENL, IR0000027, CUP: B33C22000710006); Università di Bologna.

## Conflicts of Interest

The authors declare no conflicts of interest.

## Supporting information

Materials and Methods sections; photophysical characterization; supramolecular adduct characterization; oxidation mechanism of BIH; study of **Ru** emission quenching mechanism; detection and quantification of reduction products; determination of the Apparent Quantum Yield; photostability under reaction conditions; control experiments; dynamic light scattering experiment.

## Data Availability

The data that support the findings of this study are available from the corresponding author upon reasonable request.
