# Supplementary material for: 3A Cyanide‐Bridged Ru–Fe Supramolecular Photocatalyst for Efficient CO2 Reduction
Source: ChemSusChem. 2026 Aug 13;19(16):e70969. doi: 10.1002/cssc.70969 (PMC13473391; doi:10.1002/cssc.70969)
Supplement: Supplementary file 1 [file CSSC-19-e70969-s001.pdf]

Supporting Information for

A Cyanide-Bridged Ru–Fe Supramolecular Photocatalyst for Efficient CO_2 Reduction

Francesca Mancini,[a] Alberto Bianco,[a] Albert Ruggi[b], and Giacomo Bergamini*[a]*

[a] Department of Chemistry "Giacomo Ciamician", University of Bologna, Via Piero Gobetti 85, 40129 Bologna, Italy

[b] Department of Chemistry, University of Fribourg, Chemin du Musée 9, 1700 Fribourg, Switzerland

Corresponding authors e-mail addresses: albert.ruggi@unifr.ch; giacomo.bergamini@unibo.it

1. Materials

Triethanolamine (TEOA, ≥99.5%), 2,2,2 trifluoroethanol (TFE, >99%), Acetic acid (≥99%) were purchased from *Merck* and used with no further purification. Dicyanobis(2,2'-bipyridyl)ruthenium(II) ($[Ru(bpy)_2(CN)_2] \cdot 2\ H_2O$), **Ru**, was available from previous work;[1] $[Fe(DBPy\text{-}PyA)](BF_4)_2$ (DBPy-PyA = 1-([2,2'-bipyridin]-6-yl)-N-([2,2'-bipyridin]-6-ylmethyl)-N(pyridin-2-ylmethyl)methanamine), **Fe**, was synthesized by Dr. Albert Ruggi (University of Fribourg, Switzerland),[2] 3-dimethyl-2-phenylbenzimidazoline (BIH) was synthesised from 2-Phenylbenzimidazole (purchased from *Merck*), according to the synthetic procedure reported by Beller and coworkers.[3] N_2 (filtered on *Drierite*, 99.9995%), Ar (99.9995%), and CO_2 (99.9995%) gases used for purging were supplied by *Nippon Gases*.

2. Methods

UV-Vis absorption spectra were recorded on an *Agilent Cary 300* or a *Perkin Elmer λ35* double beam spectrophotometer, using a quartz cuvette with various optical path lengths (between 0.1 cm and 1.0 cm) supplied by *Hellma*. Steady-state emission spectra were recorded on an *Edinburgh Instruments FS5* spectrofluorometer equipped with a *Hamamatsu R13456* photomultiplier tube, using a quartz 1.0 cm optical path length cuvette supplied by *Hellma*. All the emission spectra were corrected for the detector sensitivity.
Emission lifetime decays were recorded on an *Edinburgh Instruments LifeSpecII* equipped with a Time Correlated Single Photon Counting detector with Instrument Response Function (IRF) < 300 ps, a *Hamamatsu H5773-04* photomultiplier detector, using a 405 nm picosecond pulsed diode laser (EPL) as excitation source, using a quartz 1.0 cm optical path length cuvette supplied by *Hellma.* All decay traces were fitted using either monoexponential or biexponential functions. A biexponential model was applied when the **Ru–Fe** supramolecular adduct was detected and two distinct lifetimes could be resolved: a shorter lifetime attributed to the associated **Ru** center and a longer lifetime corresponding to the non-associated Ru species.
The **Ru-Fe** association process was investigated using both steady-state and time-resolved spectroscopy. The corresponding thermodynamic constants were determined through data fitting with the HypSPEC software from the HYPERQUAD suite of programs[4], which allows a global analysis of spectral data.
Transient absorption in the femtosecond range was performed by means of an Ultrafast Systems HELIOS (HE-VIS) femtosecond transient absorption spectrometer by using a Coherent Astrella Laser, as the excitation source, combined with an OPERA SOLO optical parametric amplifier (pulse width: 32 fs, 1 kHz repetition rate, selected output wavelengths: 480 nm). The Astrella system was composed of a Coherent Vitara™ that includes a modelocked Ti:Sapphire oscillator cavity pumped by the Coherent Verdi™ G-Series, a continuous-wave OPSL (optically pumped semiconductor laser) green laser, a Ti:Sa regenerative amplifier pumped by an intracavity-doubled, a DPSS (diode pumped solid state, REVOLUTION) Q-switched laser with a second-harmonic generator. Operating at 527 nm and a 1 kHz repetition rate, pump power for the regenerative amplifier module, an optical pulse stretcher, and an optical pulse compressor. The overall temporal resolution of the system is 300 fs. Air-equilibrated solutions in 0.2 cm optical path quartz cells (supplied by Hellma) were analyzed under continuous stirring. The pump energy on the sample was 8 μJ/pulse at 480 nm. Surface Xplorer V4 software from Ultrafast Systems was used for the data acquisition and analysis. The 3D data surfaces were corrected for the chirp of the probe pulse prior to the analysis. Lifetimes were taken as average

of values derived from the fitting of several decays at different wavelengths. Errors on lifetimes were estimated as average of the errors reported by the fitting software for each lifetime. Steady-state absorption spectra were registered before and after transient absorption experiments to check the photostability of the investigated system.

^{1}H NMR spectra were recorded on a *Bruker Avance III 500 MHz* instrument and the peaks are referenced to residual solvent peaks.

High-resolution mass spectra were performed in positive mode on a *Bruker FTMS Bio APEX II* with electrospray ionization (HR-ESI-MS). The compound solutions were prepared in methanol.

Attenuated total reflection Fourier Transform Infrared Spectroscopy (ATR-FTIR) measurements were performed with a *Bruker tensor II*.

H_2 and CO detection via manual reactor headspace sampling was performed on a *PerkinElmer Clarus 580* gas chromatograph equipped with a 5 Å molecular sieve column (30 m × 0.53 mm), a thermal conductivity detector (TCD), and an oven operating isothermally at 27 °C; using Argon as the carrier gas.

As observed in the stability profile (Figure 7 in Main Text), the cumulative yields of CO and H_2 display characteristic discontinuous steps or plateaus rather than a perfectly smooth kinetic curve. These variations are an artifact of the manual sampling schedule and do not reflect fluctuations in the catalyst's intrinsic activity. While the photoreactor remained under constant, continuous light irradiation for the entire duration of the test, headspace sampling was performed manually and was suspended during overnight periods. During these unmonitored night hours, photocatalytic product evolution proceeded uninterrupted, leading to the accumulation of gases within the sealed headspace. The apparent sharp increases or jumps in the yield curve represent the cumulative gas volume measured immediately upon the resumption of manual sampling the following morning.

Cyclic voltammetry was performed with a Gamry Interface 1000B potentiostat in a three-electrode cell by using a glassy carbon working electrode, a platinum wire as counter electrode and a silver wire as pseudo reference electrode. Potentials were calibrated versus Saturated Calomel Electrode (SCE) by addition of ferrocene (E = + 0.41 V vs SCE) at the end of the experiments. Tetrabutylammonium hexafluorophosphate ($TBAPF_6$, 0.1 M in acetonitrile) was used as supporting electrolyte.

The evaluation of nanoparticle formation in solution was carried out by Dynamic Light Scattering measurements on a *LSI - NanoLab 3D*; the samples were housed in quartz cuvettes of 1.0 cm optical path length.

3. Photophysical characterization

Absorption and emission spectra of Ru complex

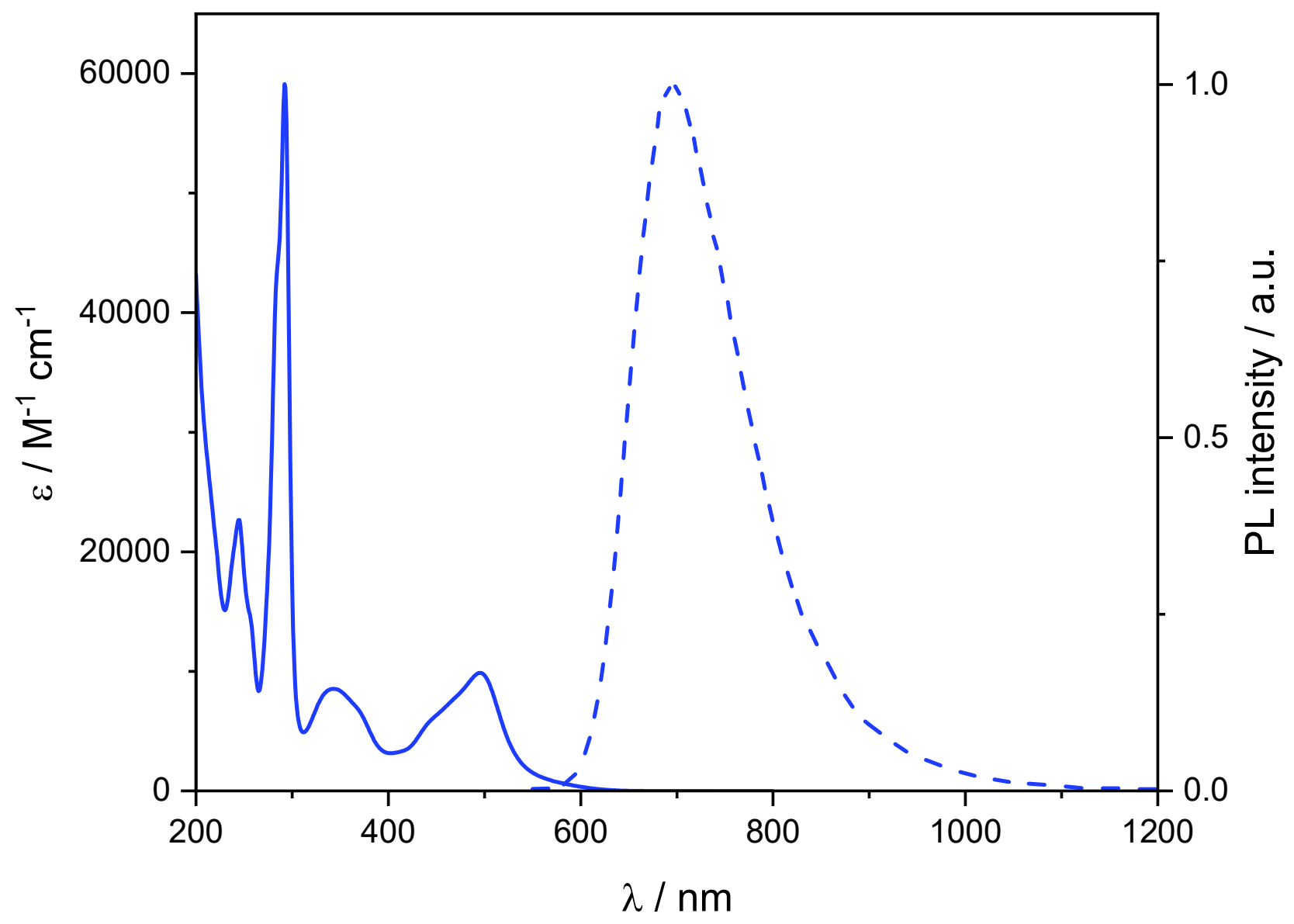

Figure S1. Absorption (solid line) and emission (dashed line, λ_{exc} = 496 nm) spectrum of $[Ru(bpy)_2(CN)_2]$ in acetonitrile solution.

Absorption spectrum of Fe complex

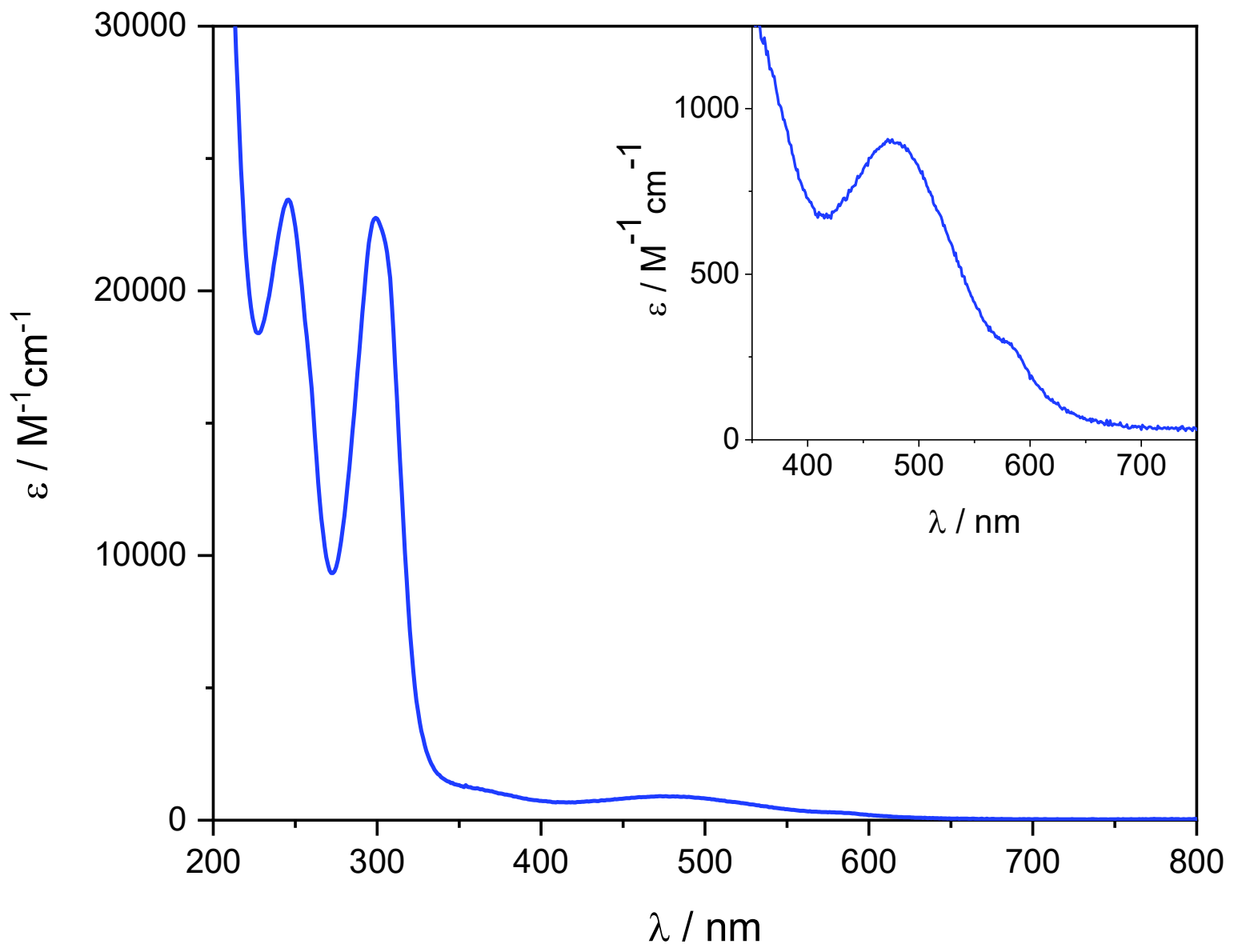

Figure S2. Absorption spectrum of $[Fe(DBPy\text{-}PyA)](BF_4)_2$ in acetonitrile solution; inset: expanded view of the d-d band transition centered at 476 nm.

4. Supramolecular adduct characterization

HR-ESI-MS measurements

A 1:1 solid-state mixture of the ruthenium and iron complexes was prepared by thoroughly grinding the two crystalline solids in a mortar and pestle. The mechanochemically activated mixture was then dissolved in methanol and analyzed by high-resolution electrospray ionization mass spectrometry (HR-ESI-MS).

An expanded view of the relevant region reveals a dominant signal at *m/z* = 483, assigned to the $[Ru–Fe]^{2+}$ supramolecular adduct. The monoisotopic masses of the individual complexes were determined to be 465.48 (**Ru** complex) and 500.39 (**Fe** complex, corrected for the loss of the labile coordination solvent). The vacant coordination site on the iron center allows binding of the cyano ligand of the Ru complex. Considering the sum of the monoisotopic masses and the 2+ charge of the resulting heterometallic species, the calculated *m/z* value matches the experimentally observed peak at 483, supporting the assignment.

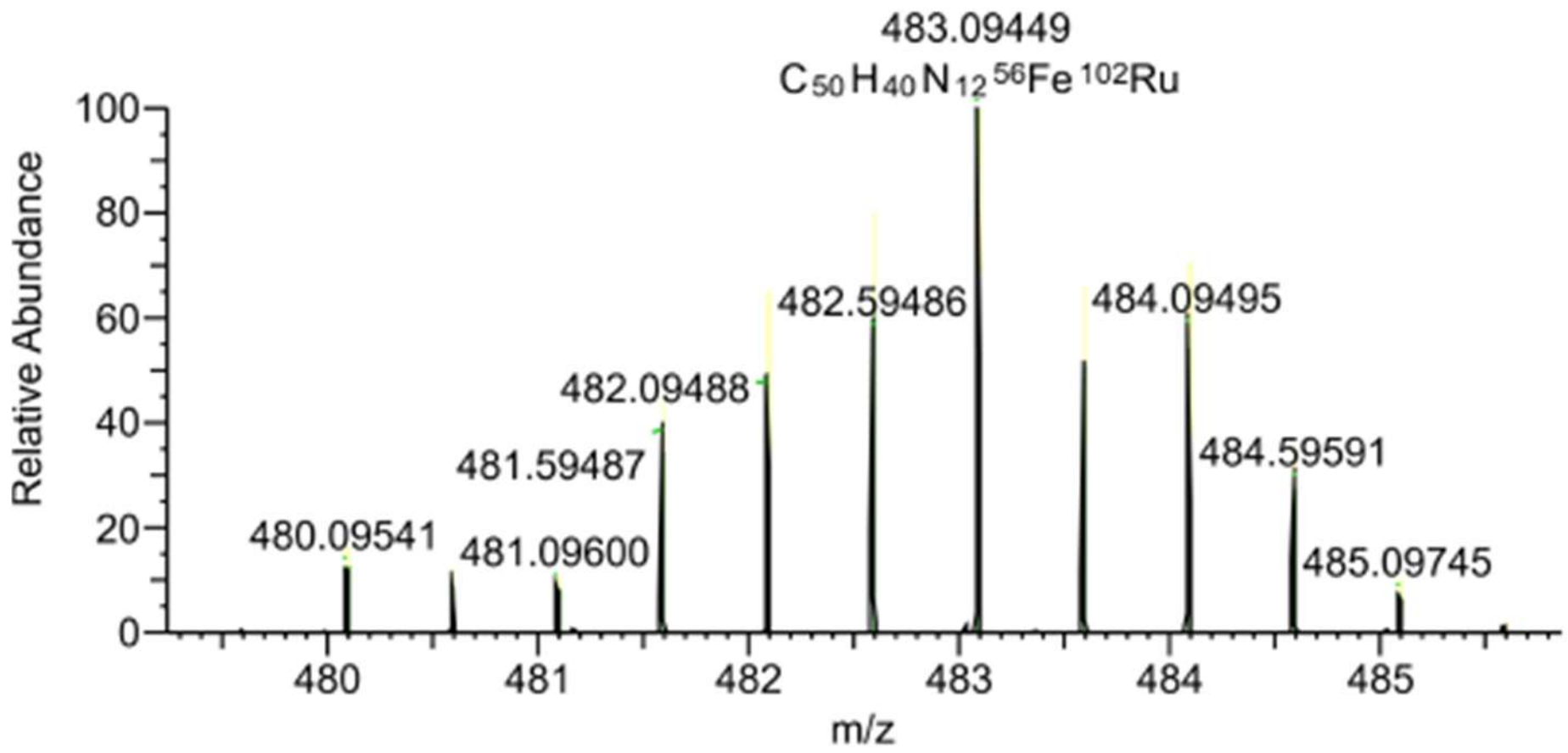

Figure S3. HR-ESI-MS spectrum of a methanol solution of **Ru–Fe** 1:1 supramolecular adduct.

ATR-FTIR measurement

ATR-IR spectroscopy also provides compelling evidence for the formation of the **Ru–Fe** adduct. The vibrational modes of the cyanide ligands serve as sensitive diagnostic probes, as their stretching frequencies are influenced by electronic delocalization effects arising from the coordination of Lewis acids to the nitrogen atom of the cyano group.

Focusing on the spectral region between 2150 and 2000 cm^{-1}, where the cyano stretching bands are typically observed, the IR spectrum of the **Ru** complex displays a broadened feature comprising two distinct components (indicated by the black arrows in figure S4). A primary maximum at 2054 cm^{-1} and a secondary shoulder at 2065 cm^{-1} can be attributed to the out-of-phase and in-phase stretching vibrations of the two cyano ligands, respectively.

Upon formation of the **Ru–Fe** adduct, these two peaks shift to higher wavenumbers (2056 and 2070 cm^{-1}, respectively), a behavior commonly associated with Lewis acid coordination to the cyano ligand. Such interaction results in a lowering of the CN π-bonding orbital energy, thereby increasing the stretching frequency.[5]

This spectroscopic response has been previously reported by Bignozzi and Scandola in a study on heterometallic complexes featuring $[Ru(bpy)_2(CN)_2]$ centers coordinated to one or two $[Pt(dien)]^{2+}$ units.[6]
A similar trend is also evident in the work of Wenger and coworkers,[5] who investigated the effects of borylation on the cyanide ligands of $[Ru(bpy)_2(CN)_2]$. In that case, conversion to the corresponding isocyanoborate complex similarly resulted in a shift of the CN stretching modes to higher energies.

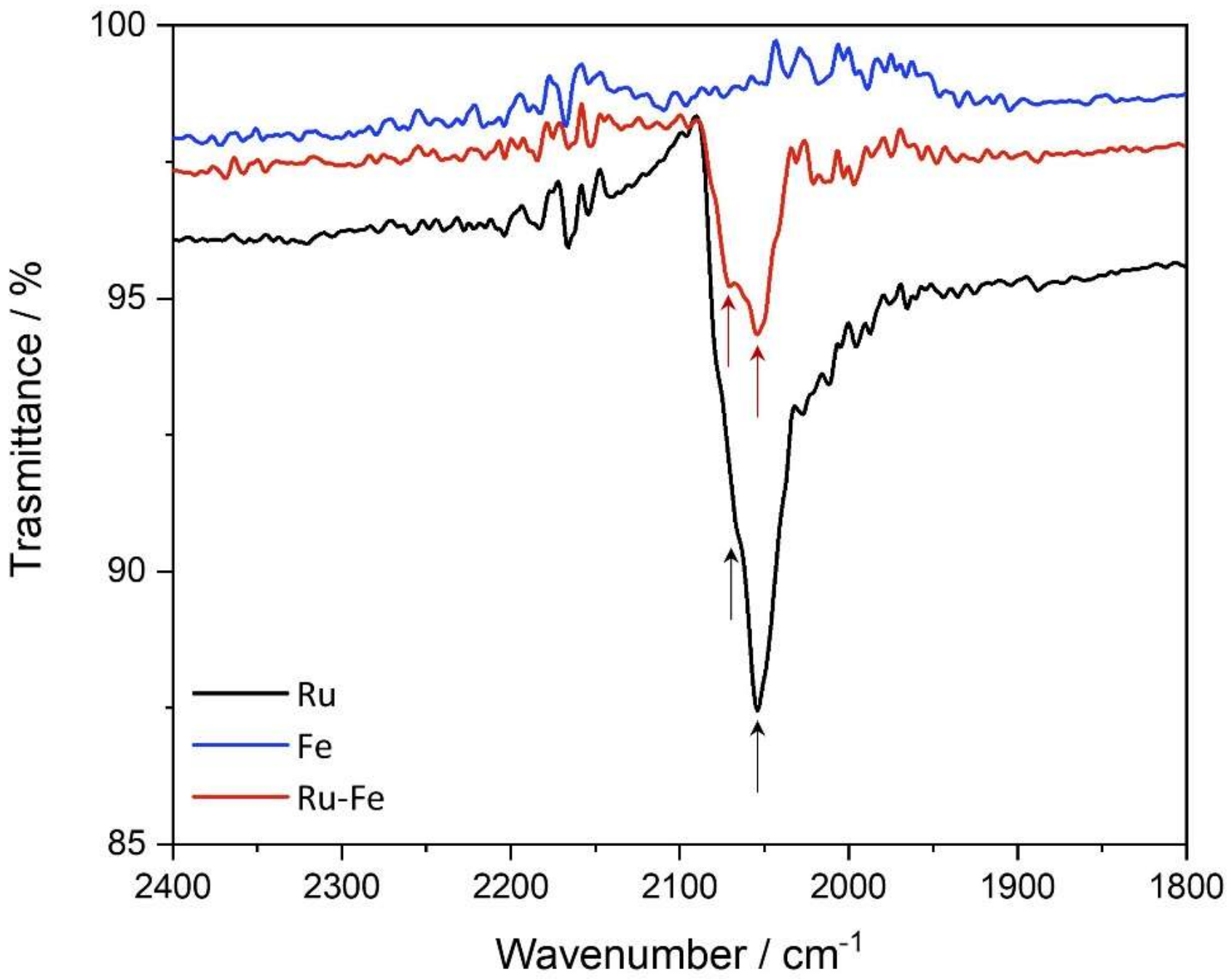

Figure S4. Magnified view of the ATR-FTIR spectrum of **Ru** (black curve), **Fe** (blue curve) and **Ru–Fe** adduct (red curve).

5. Oxidation mechanism of BIH

3-dimethyl-2-phenylbenzimidazoline functions as a two-electron/one-proton (hydride) donor. After the first oxidation step, the proton of $BIH^{\bullet+}$ becomes very acidic and needs to be captured by a base to prevent back electron transfer between $BIH^{\bullet+}$ and PS.[7]

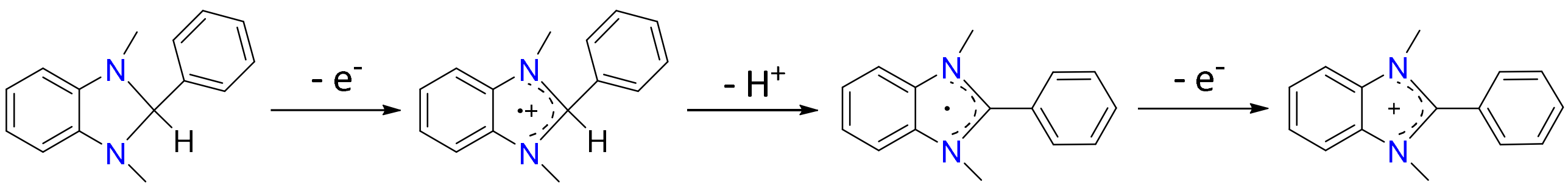

Figure S5. Two-electron and one-proton BIH oxidation mechanism.

6. Ru emission quenching: a mechanism study

Ultrafast transient absorption spectroscopy: Fe^{-} species detection

Since the pre-association between the photosensitizer and catalyst has been proven, ultrafast transient absorption spectroscopy was performed to probe the excited-state dynamics of the **Ru–Fe** assembly. The measurements confirm an oxidative quenching pathway, with sub-picosecond electron transfer from photoexcited **Ru** to the **Fe** center, leading to the formation of a reduced Fe^{-} intermediate. This species decays with a lifetime of approximately 9 ps.

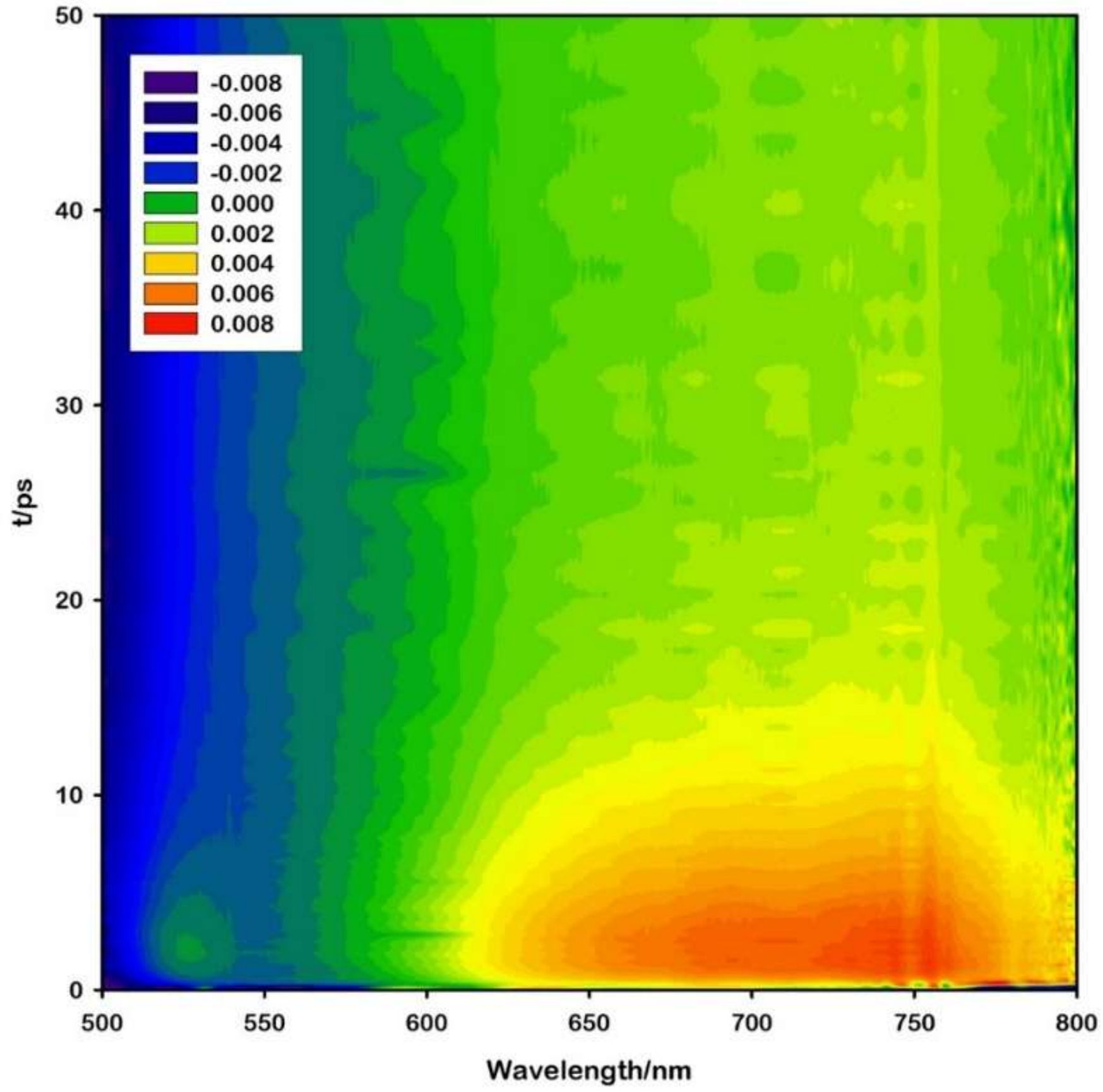

Figure S6. Ultrafast transient absorption map of the **Ru–Fe** supramolecular assembly showing formation and decay within 20 ps of the reduced **Fe^{-}** species, inset shows the ΔAbs color-coding.

BIH role as Ru emission quencher

Upon addition of 0.1 M BIH to the **Ru–Fe** supramolecular adduct solution, a dynamic quenching process was observed in addition to the previously identified static quenching. Specifically, the lifetime of the free Ru complex decreased from 65 ns to 22 ns, indicating that BIH is able to reductively quench its excited state.

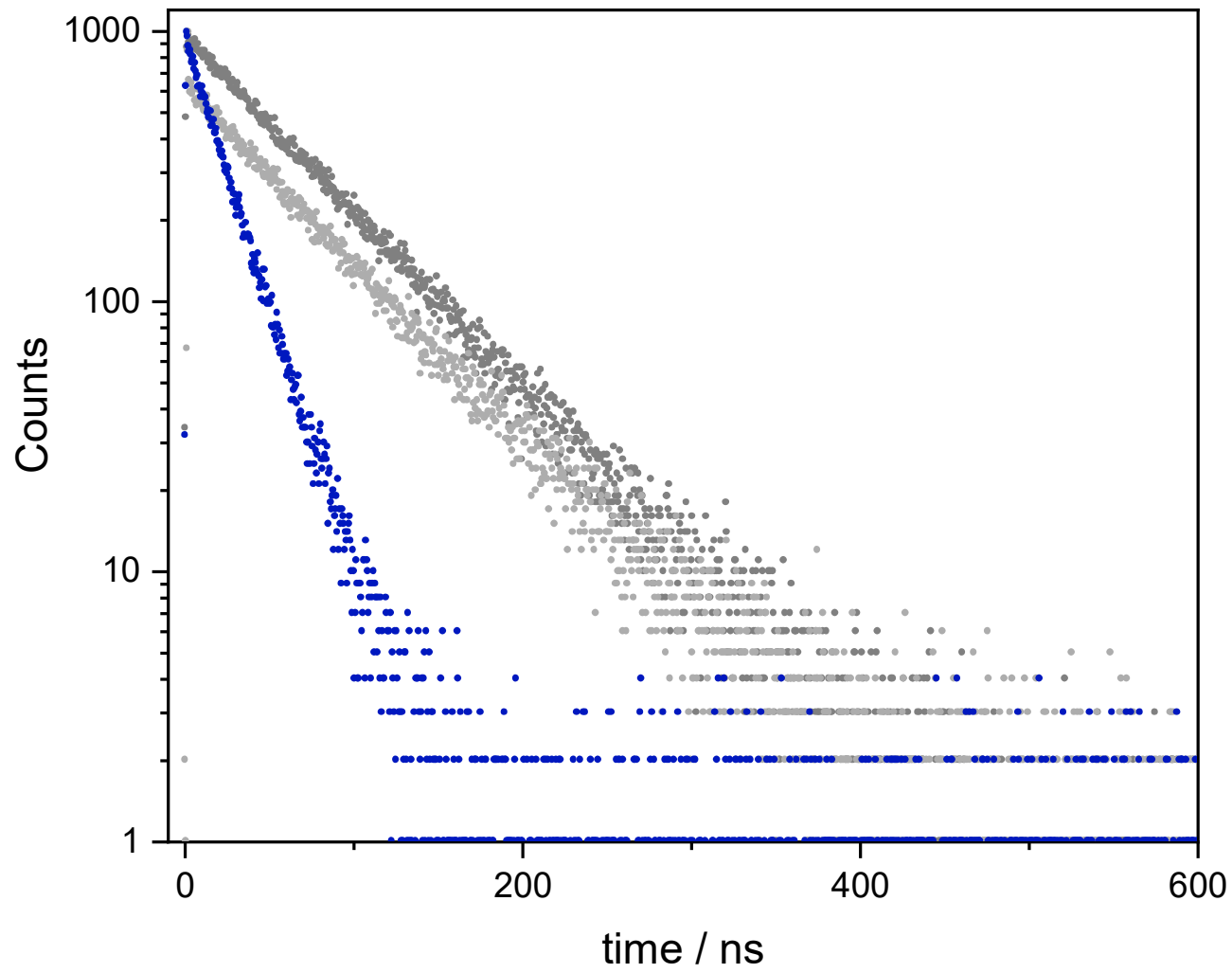

Figure S7. Luminescence intensity decays showing the effects of BIH addition to the **Ru–Fe** supramolecular adduct; Ru (grey curve), **Ru–Fe** (light grey curve), **Ru–Fe** + BIH (blue curve).

Steady-state absorption and emission measurements were carried out to investigate the interaction between BIH and the excited state of the ruthenium complex (Figure S8). A diluted **Ru** solution was prepared to maintain an absorbance <0.1 at the ^{1}MLCT band maximum (496 nm), ensuring linearity between absorbance and emission intensity. Spectral changes were monitored upon successive additions of a BIH solution (100 mM) to 2.5 mL of **Ru** complex in acetonitrile (9.2 µM), up to 28 mM (696 µL added). In the absorption spectra, a slight increase of the MLCT band is observed up to 6 mM of BIH, followed by a gradual decrease at higher additions due to the dilution effect. Emission spectra recorded at λ_{exc} = 496 nm show a progressive decrease in phosphorescence intensity with increasing BIH concentration. The Stern–Volmer plot (Figure S8b) displays a linear trend over the full concentration range, yielding a bimolecular quenching constant $k_q = 3.7 \times 10^5\ M^{-1}\ s^{-1}$. These data, together with the lifetime data reported above, confirm that BIH acts as a dynamic quencher of the ***Ru** excited state.

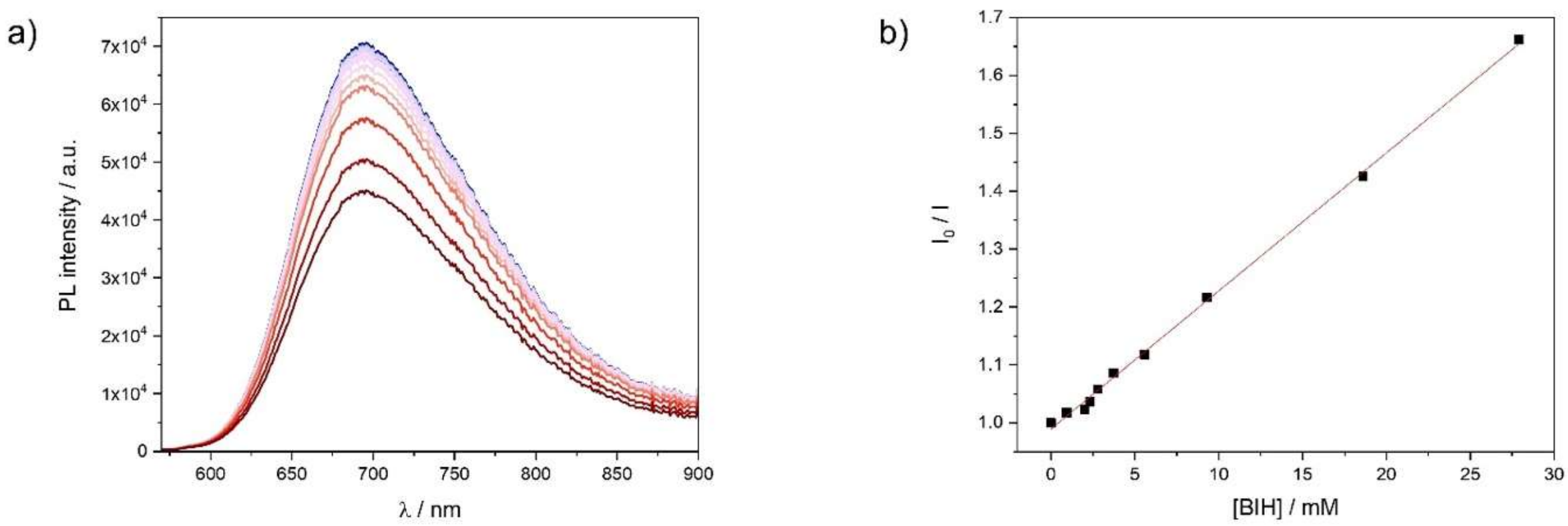

Figure S8. a) Steady-state emission (λ_{exc} = 496 nm) spectra showing interaction between Ru and increasing concentrations of BIH (from 0 to 28 mM), b) Stern-Volmer plot.

TEOA role as Ru emission quencher

Time-correlated single photon counting measurements were repeated upon addition of 0.3 M TEOA to the acetonitrile solution already containing the **Ru–Fe** adduct and 0.1 M BIH. The addition of TEOA results in a negligible lifetime change.

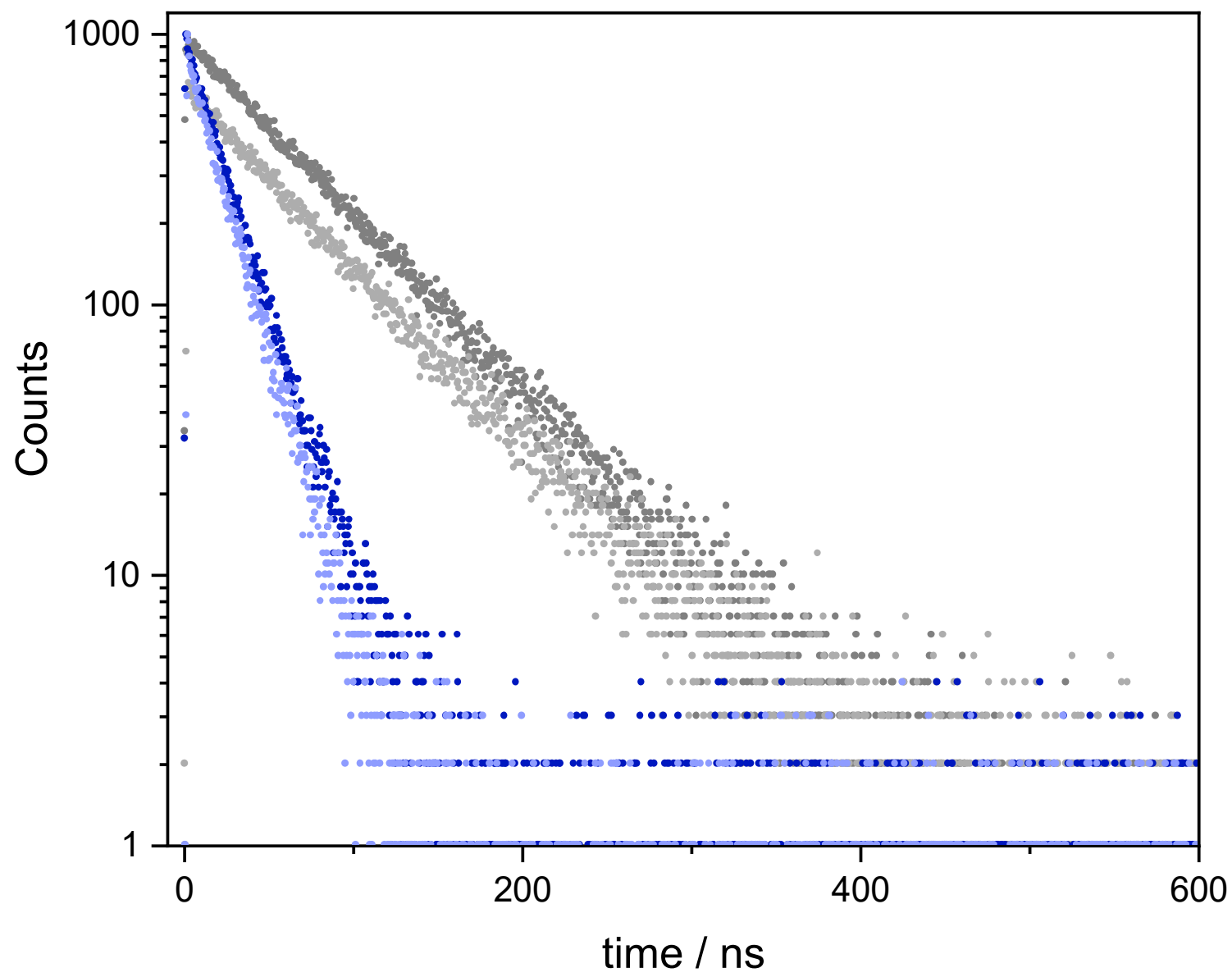

Figure S9. Luminescence intensity decays showing the effects of individual component additions to the photocatalytic system, with particular emphasis on the role of TEOA (light blue curve); **Ru** (grey curve), **Ru–Fe** (light grey curve), **Ru–Fe** + BIH (blue curve) and **Ru–Fe** + BIH + TEOA (light blue curve).

To confirm unambiguously that TEOA does not quench the **Ru** excited state, both absorption and emission spectra were recorded for an acetonitrile solution of **Ru** (232 μM) upon addition of TEOA (0.3 M), in a total volume of 3 mL (Figure S10). In the absorption spectrum, a slight blue shift of the MLCT band is observed, which is attributed to a possible change in solvent polarity caused by the high concentration of TEOA. The emission spectra of Ru alone and with TEOA show no excited-state quenching processes.

The absence of emission quenching provides strong evidence that dynamic quenching via electron transfer from TEOA to the excited state of Ru is not thermodynamically favorable under operating conditions.

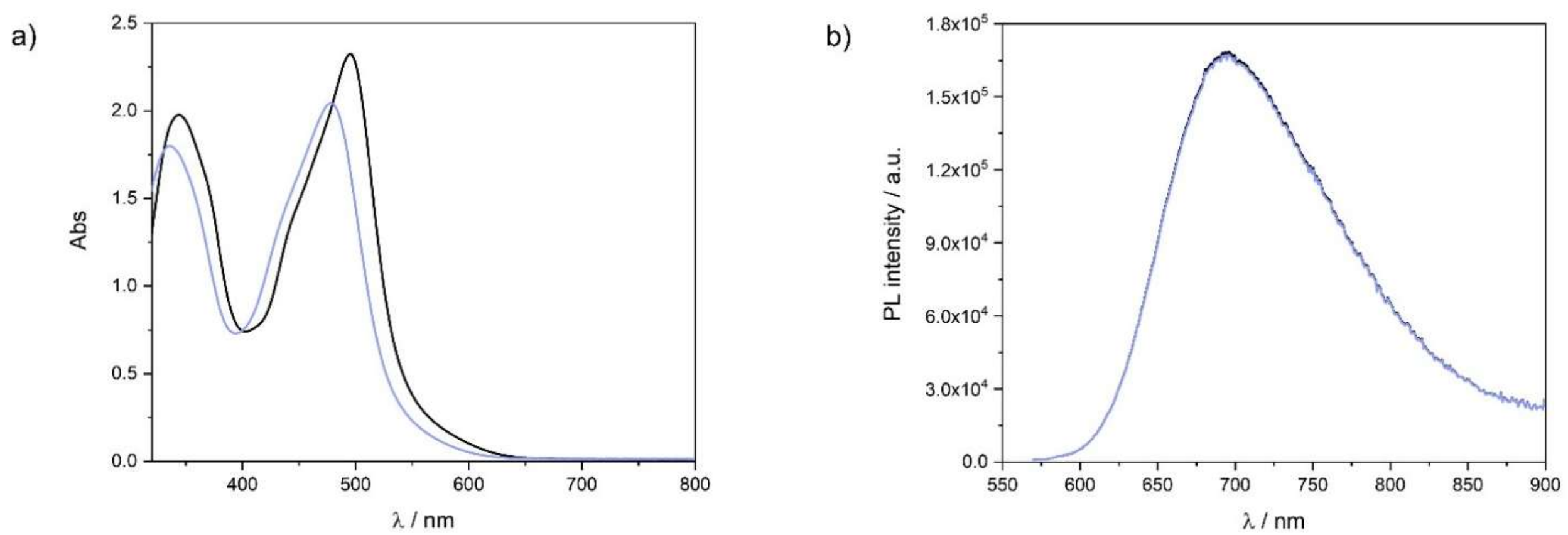

Figure S10. a) Absorption and b) emission (λ_{exc} = 475 nm) spectra highlighting the interaction between **Ru** (black curve) and TEOA (light blue curve).

Evidence for CO_2 coordination

Further evidence for the interaction between CO_2 and the **Ru–Fe** complex was obtained from two independent experiments. First, the spectrophotometric test previously reported in Fig. 6 was repeated in the absence of TEOA (Fig. S11). The same spectral variations were observed, demonstrating that the response originates from the interaction of CO_2 with the **Fe** catalytic center rather than from polarity changes induced by TEOA in solution.

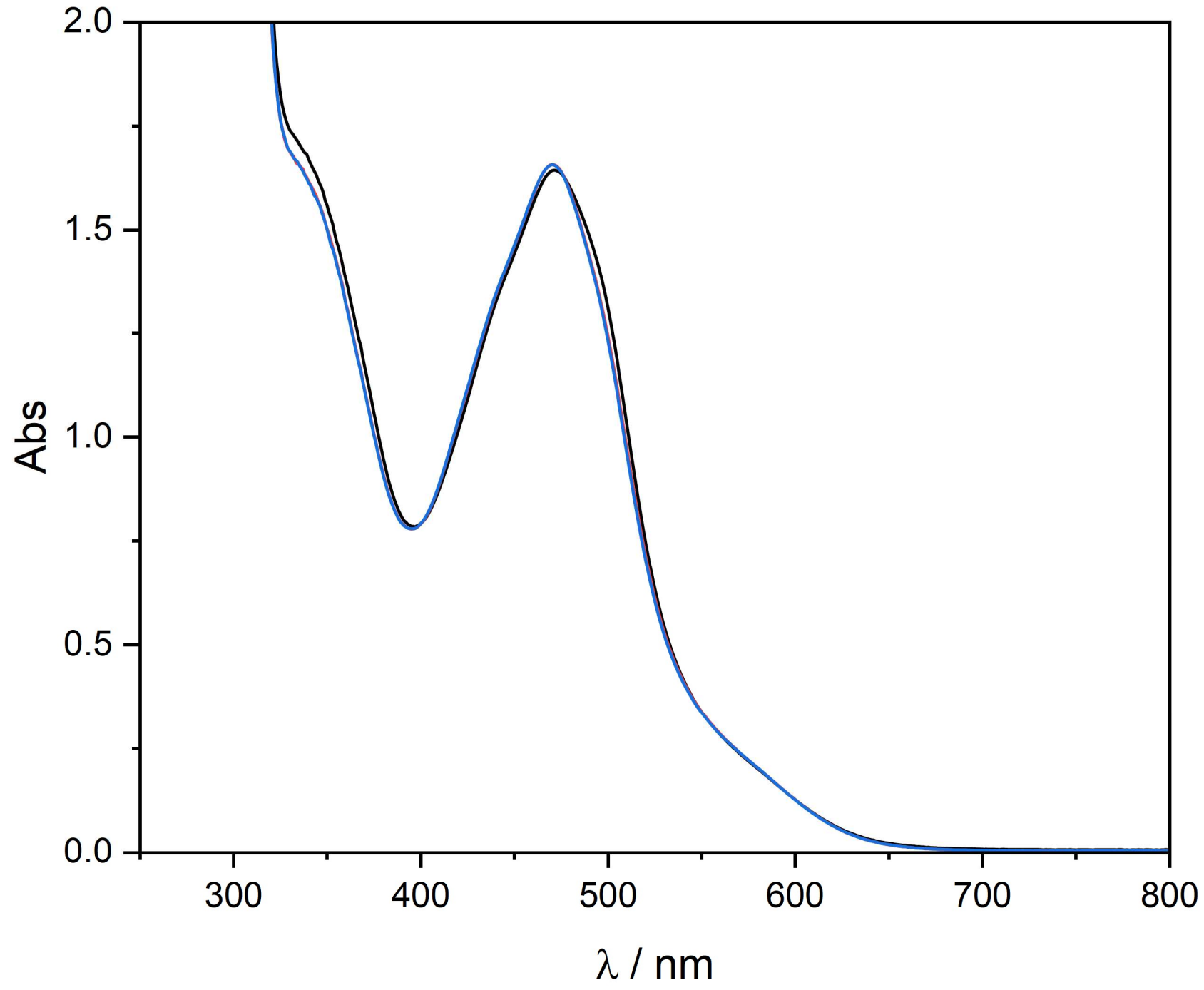

Figure S11. Reversible absorption spectral changes of the preassembled **Ru–Fe** supramolecular complex upon CO_2 binding in the absence of TEOA; **Ru–Fe** (black curve), **Ru–Fe** after CO_2 bubbling (red curve), **Ru–Fe** after argon purging (blue curve).

Additional evidence for CO_2 coordination was provided by cyclic voltammetry measurements (Figure S12). Under argon atmosphere (black trace), the **Ru–Fe** complex displays three distinct reduction peaks at – 1.51, − 1.65 and − 1.89 V vs SCE. Upon introduction of CO_2 (red trace), the first reduction peak, ascribable to iron center reduction within the supramolecular adduct, shifts from − 1.51 to − 1.53 V vs SCE. When argon was subsequently reintroduced into the system (blue trace), the original voltammetric profile was largely recovered, confirming the reversible nature of the interaction. No changes are observed for the reduction processes centered on **Ru** subunit.

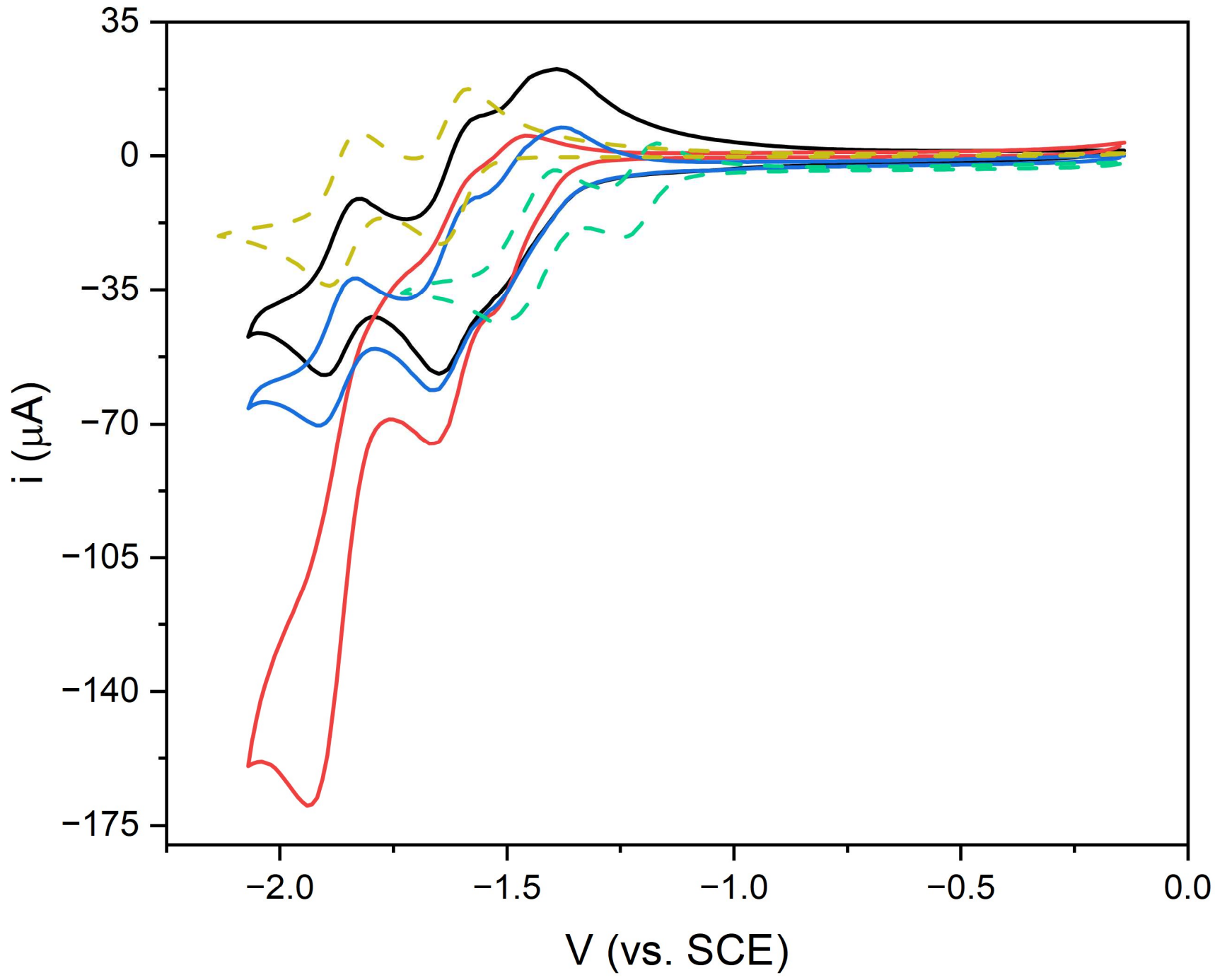

Figure S12. Cyclic voltammetry curves as evidence of CO_2 coordination to **Ru–Fe** complex, formed in situ by mixing 1.87 mg (4.0 μmol) of **Ru** and 2.26 mg (3.2 μmol) of **Fe** in 3 mL of 0.1 M $TBAPF_6$ solution in acetonitrile**. Ru–Fe** under Ar atmosphere (black curve), **Ru–Fe** after CO_2 bubbling (red curve), and **Ru–Fe** after argon purging (blue curve). To make the assignation of the observed reduction processes easier, cyclic voltammogram of separated **Ru** and **Fe** under Ar atmosphere (dashed yellow and green curve, respectively) also also reported.

7. Detection and quantification of reduction products

Gaseous products

The gaseous products, namely hydrogen (H_2) and carbon monoxide (CO), were detected and quantified using gas chromatography (GC). Reactor headspace gas analysis was carried out by withdrawing 50 μL samples once every hour, using a Hamilton gas-tight sample lock syringe, which were then injected into the GC system. Calibration of the GC for quantitative analysis was performed using H_2/Ar and CO/Ar gas mixtures. These standard mixtures were prepared with an automated gas mixing apparatus and stored in gas sampling bags. Known volumes were sampled with the same Hamilton syringe and injected into the GC under identical conditions. The integrated area under the corresponding H_2 and CO peaks was recorded. From the known composition of the calibration mixtures, the moles of each gas were first calculated using the sampled volume and the ideal gas law:

$$n = \frac{PV}{RT} \tag{1}$$

where P is supposed to be 1 atm, R is the molar gas constant and T = 293.15 K.

Subsequently, calibration curves were constructed by plotting the number of moles against the corresponding peak areas, and linear regression was applied to obtain the calibration curve equation.

For experimental samples, the number of moles of H_2 and CO in each 50 µL headspace aliquot was determined using these calibration curves. Finally, these values were scaled by a factor of 200 to account for the total headspace volume of 10 mL within the photoreactor.
The photocatalytic efficiency of the system with respect to hydrogen and carbon monoxide production was assessed by calculating the turnover number (TON), which quantifies the number of product molecules generated per catalyst molecule. The TON is defined by Equation 2.

$$TON = \frac{n_{product}}{n_{catalyst}} \quad (2)$$

Where $n_{catalyst}$ are the total moles of **Fe** added to the system n_{Fe}.

Soluble products

Formate quantification was carried out using ¹H NMR spectroscopy, with the formate proton signal appearing at δ = 8.3 ppm. Following irradiation, the reaction mixture was adjusted to basic pH by the addition of the minimum necessary amount of NaOH. The solution is then concentrated to dryness under reduced pressure. The resulting solid residue was re-dissolved in 1.5 mL of deuterium oxide (D_2O) and filtered, after which 1.2 mL of the solution was transferred into an NMR tube for analysis; 2 µL of dimethylformamide were added to the D_2O solution as an internal standard.[8]
Typically, the singlet centered at 7.92 ppm, corresponding to the DMF formyl proton, is used for quantification, and it integrates for one proton. However, in our system, this signal was difficult to resolve due to overlap with multiplets likely arising from BIH degradation products. Consequently, the singlet at 3.03 ppm, attributed to the methyl protons of DMF, was employed as the reference signal for quantification.
A representative example of the ¹H NMR spectra used for formate detection is shown in Figure S13.

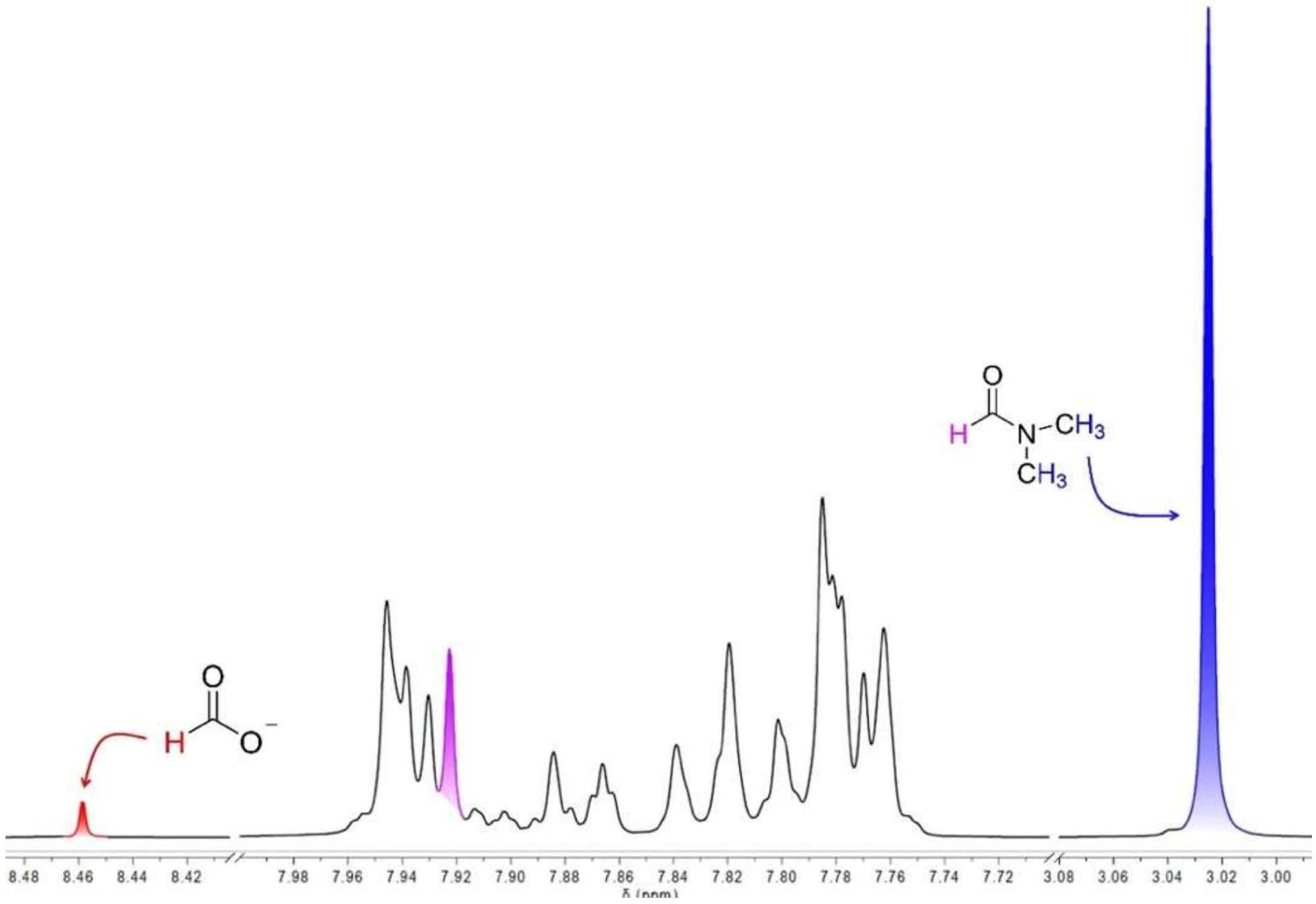

Figure S13. Zoom of the ¹H NMR spectrum of the irradiated photocatalytic system (**Ru** 232 µM, **Fe** 186 µM, BIH 0.1 M, TEOA 0.3 M). In red, the formate peak; in blue, three-proton integrating DMF peak used as internal standard, in purple one-proton integrating DMF peak typically employed as reference for formate quantification.

8. Determination of the Apparent Quantum Yield

The photocatalytic efficiency of the system is further assessed by determining the apparent quantum yield (AQY). This parameter is defined as the ratio between the CO evolved moles multiplied by the electrons involved in the targeted reaction and the moles of incident photons per unit of time (equation 3).

$$AQY(\%) = \frac{(\text{number of reacted electrons} \times n_{CO}) \times s^{-1}}{\text{einstein} \times s^{-1}} \times 100 \quad (3)$$

Since the photoreduction of CO_2 to CO involves a two-electron process, the numerator corresponds to twice the moles of CO evolved. The photon flux (moles of photons per unit time) is determined by chemical actinometry.

For this purpose, a 3 mL acetonitrile solution of $[Ru(bpy)_3]^{2+}$ in the presence of 1,9-diphenylanthracene (DPA) is employed as the actinometer, using the same optical configuration as in the photocatalytic experiments. The actinometric reaction monitored is the well-established photooxidation of DPA to its corresponding endoperoxide by $[Ru(bpy)_3]^{2+}$, according to the procedure reported by Scaiano and coworkers.[9] Based on this measurement, the incident photon flux at the photoreactor surface is determined to be 1.32×10^{-8} mol s^{-1}. Considering a total CO production of 107 µmol after 25 h of irradiation, the apparent quantum yield of the system is calculated to be 18%.

9. Photostability under reaction conditions

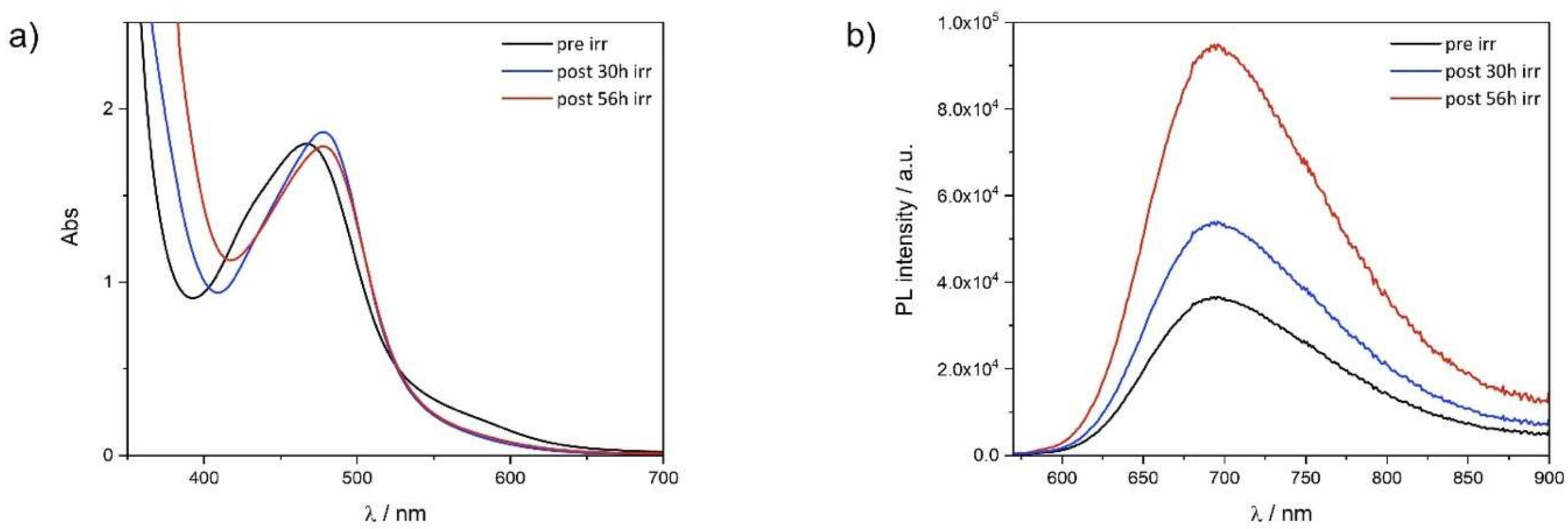

Figure S14. a) Absorption and b) emission (λ_{exc} = 475 nm) spectra of the **Ru** initial complex and the optimized system (**Ru** 232 µM, **Fe** 185 µM, BIH 0.1 M and TEOA 0.3 M, red curves) after 30 hours (I cycle, black curves) and 56 hours of irradiation (II cycle, blue curves) with a 475 nm LED.

10. Control experiments

CO_2-free control system

Spectral variations in absorption and emission observed after 30 hours of irradiation of the system saturated with argon, i.e. in the absence of CO_2, indicate degradation of the system. This behaviour is consistent with the hypothesis that the production of formate observed under CO_2-free conditions arises from the degradation of system components, as discussed in the main text.

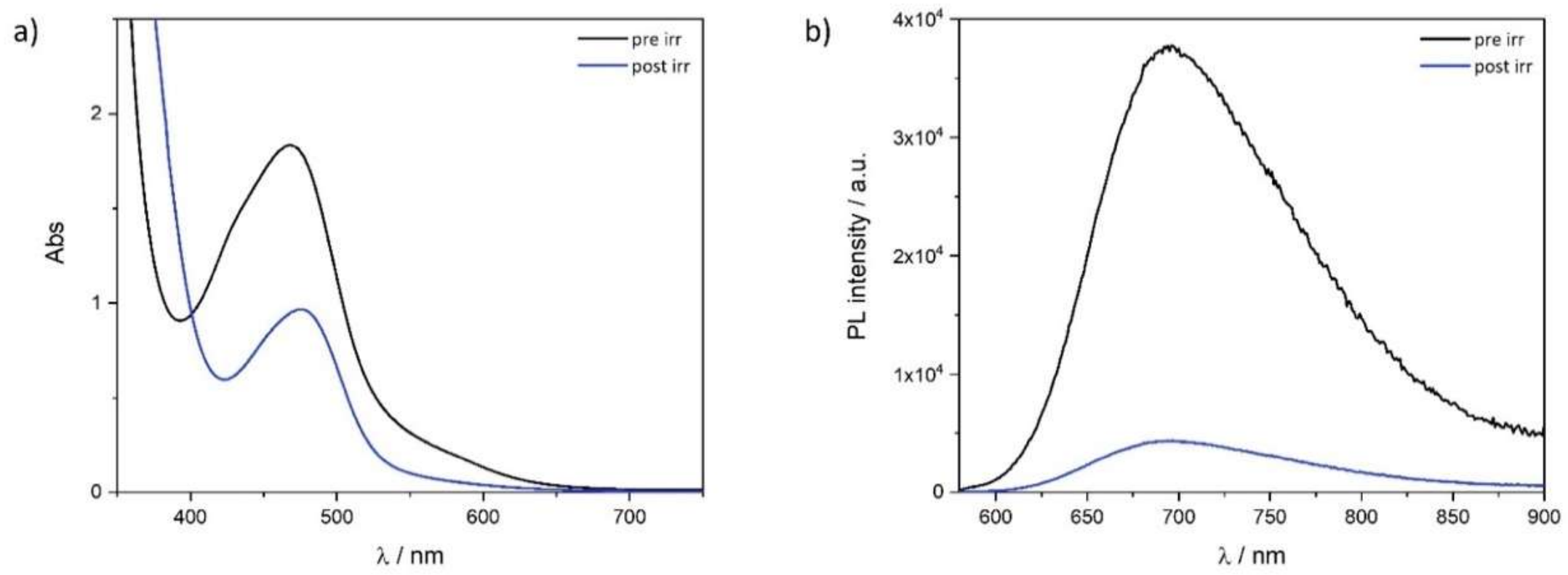

Figure S15. a) Absorption and b) emission spectra of the photocatalytic system in the absence of CO_2, before and after the irradiation. Acetonitrile solution of **Ru** 232 μM, **Fe** 186 μM, BIH 0.1 M, TEOA 0.3 M. Irradiation with 475 nm LED for 30 hours.

Control system with $Fe(BF_4)_2 \cdot 6\ H_2O$ as catalyst

The absorption spectra of the system employing an iron salt, iron(II) tetrafluoroborate hexahydrate ($Fe(BF_4)_2 \cdot 6\ H_2O$), as the catalytic component, are presented below. In Figure S16, a pronounced hypsochromic shift of the Ru-based ^{1}MLCT band is observed, with the absorption maximum shifting from 496 to 420 nm. This substantial spectral variation is indicative of a strong interaction between the Ru complex and the iron salt, suggesting efficient adduct formation.[10] Notably, the extent of this interaction appears to be greater than that observed with the iron complex, likely due to the complex's higher steric hindrance. Upon the addition of 0.1 M BIH, an alteration in the shape of the Ru ^{1}MLCT band is observed, characterized by the emergence of a shoulder at 498 nm. However, spectral acquisition repeated after five minutes shows complete recovery of the original band shape and position. The subsequent addition of TEOA induces a blue shift of the ^{1}MLCT band, further supporting the sensitivity of the system to its chemical environment. After 30 hours of irradiation, a significant decrease in the absorbance of the Ru ^{1}MLCT band is evident, pointing to limited photostability of the system under these conditions.

These findings underscore the critical role of the ligand scaffold, not only as a structural element facilitating electron transfer during photocatalysis, but also as a key factor in determining the overall stability and robustness of the catalytic system.

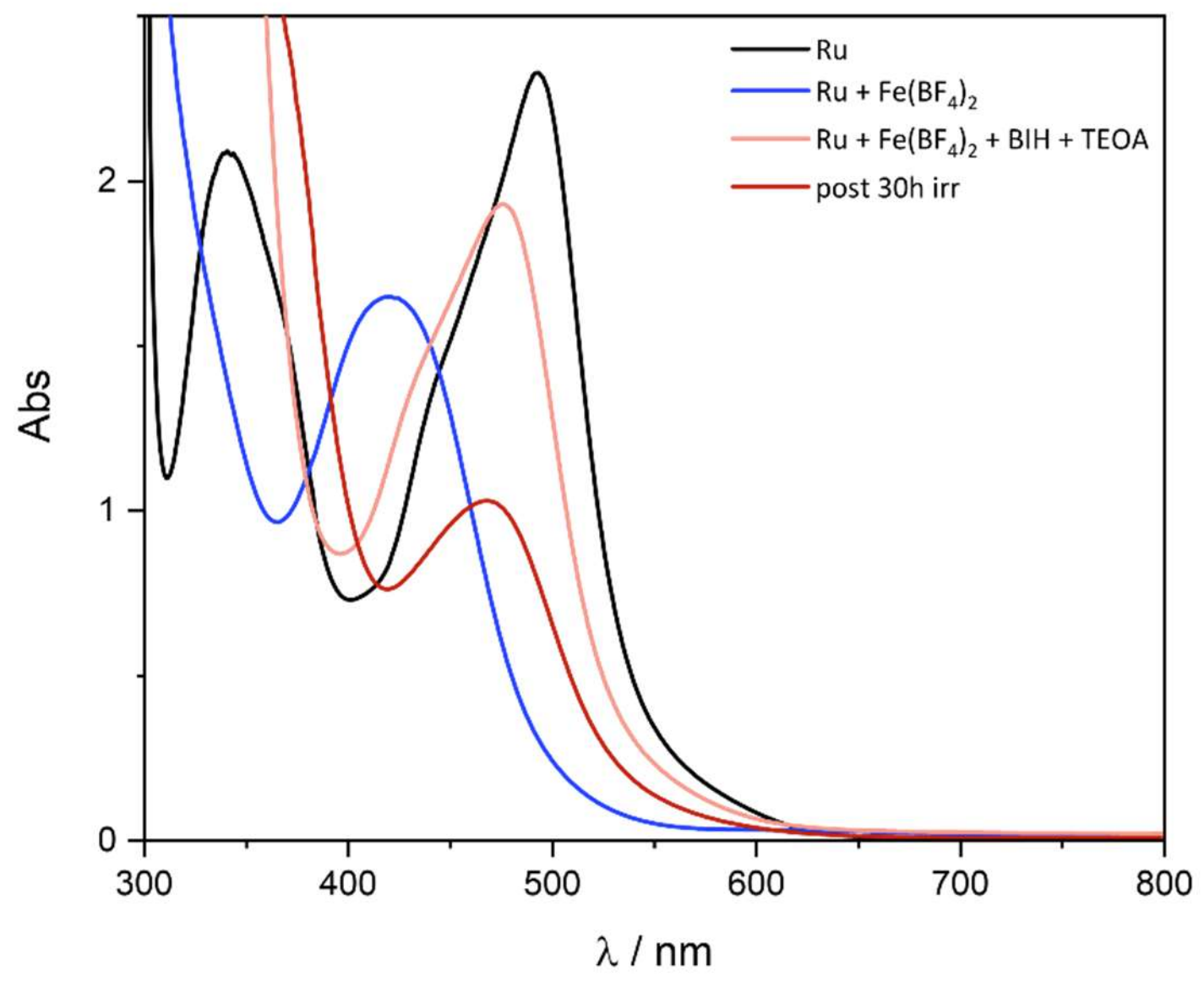

Figure S16. Absorption spectra of the photocatalytic system in which the **Fe** complex was replaced by iron(II) tetrafluoroborate hexahydrate.

Products (H_2 and $HCOO^-$) detection

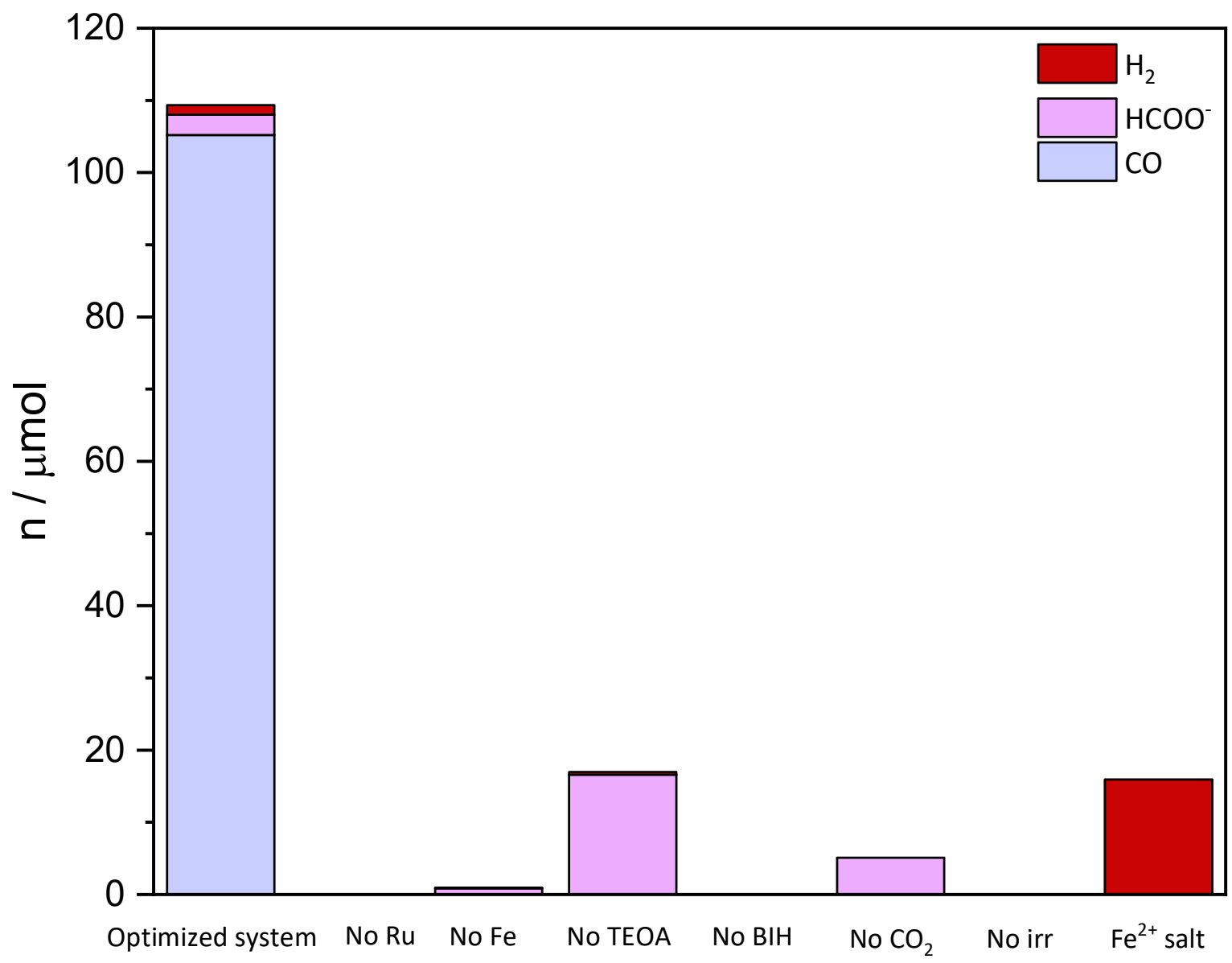

Figure S17. H_2 and $HCOO^-$ production over 30 hours of 475 nm LED irradiation of all the control systems (Fe^{2+} salt is $Fe(BF_4)_2 \cdot 6\ H_2O$).

11. Dynamic light scattering

Dynamic light scattering (DLS) measurements were performed after 30 hours of irradiation of an acetonitrile solution containing 232 µM **Ru**, 186 µM **Fe**, and 0.1 M BIH at 20 °C. The correlation function showed no detectable light-scattering events in the irradiated photocatalytic mixture, indicating that no nanoparticles were formed under the catalytic conditions.

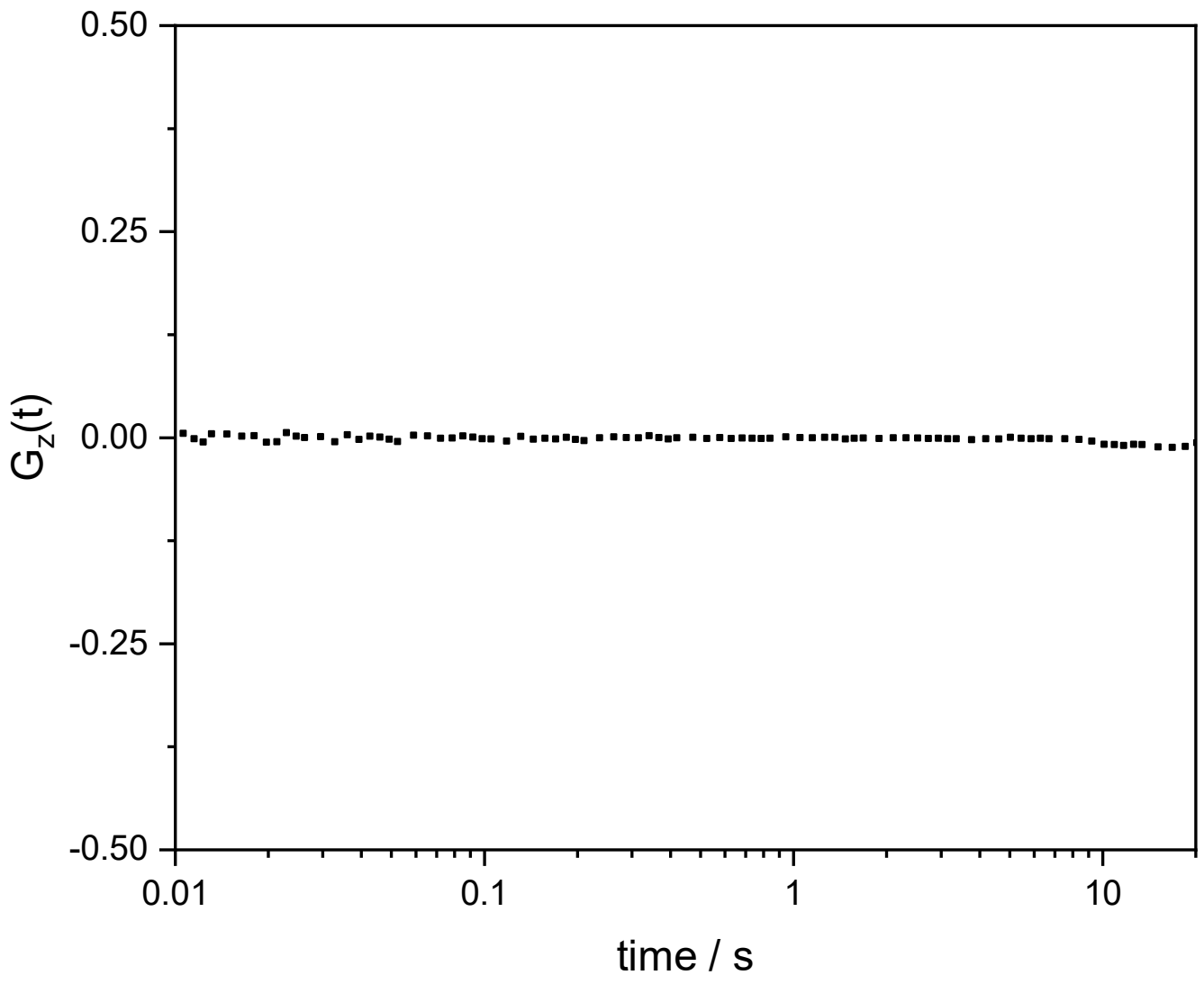

Figure S18. Correlation function from DLS measurement after 30 h of irradiation of an acetonitrile solution containing 232 µM of **Ru**, 186 µM of **Fe**, and 0.1 M of BIH at 20°C.

References

(1) Barigelletti, F.; Juris, A.; Balzani, V.; Belser, P.; Von Zelewsky, A. Temperature Dependence of the Bis(2,2'-Bipyridine)Dicyanoruthenium(II) and Bis(2,2'-Bipyridine)(2,2'-Isobiquinoline)Ruthenium(II) Luminescence. *J. Phys. Chem.* **1987**, *91* (5), 1095–1098. https://doi.org/10.1021/j100289a018.

(2) Droghetti, F.; Amati, A.; Pascale, F.; Crochet, A.; Pastore, M.; Ruggi, A.; Natali, M. Catalytic CO2 Reduction with Heptacoordinated Polypyridine Complexes: Switching the Selectivity via Metal Replacement. *ChemSusChem* **2024**, *17* (4), e202300737. https://doi.org/10.1002/cssc.202300737.

(3) Rosas-Hernández, A.; Steinlechner, C.; Junge, H.; Beller, M. Earth-Abundant Photocatalytic Systems for the Visible-Light-Driven Reduction of CO2 to CO. *Green Chem.* **2017**, *19* (10), 2356–2360. https://doi.org/10.1039/C6GC03527B.

(4) Gans, P.; Sabatini, A.; Vacca, A. Investigation of Equilibria in Solution. Determination of Equilibrium Constants with the HYPERQUAD Suite of Programs. *Talanta* **1996**, *43* (10), 1739–1753. https://doi.org/10.1016/0039-9140(96)01958-3.

(5) Schmid, L.; Kerzig, C.; Prescimone, A.; Wenger, O. S. Photostable Ruthenium(II) Isocyanoborato Luminophores and Their Use in Energy Transfer and Photoredox Catalysis. *JACS Au* **2021**, *1* (6), 819–832. https://doi.org/10.1021/jacsau.1c00137.

(6) Bignozzi, C. A.; Scandola, F. Cyano-Bridged Ruthenium(II)/Platinum(II) Complexes: Synthesis, Photophysical Properties, and Excited-State Redox Behavior. *Inorg. Chem.* **1984**, *23* (11), 1540–1545. https://doi.org/10.1021/ic00179a015.

(7) Pellegrin, Y.; Odobel, F. Sacrificial Electron Donor Reagents for Solar Fuel Production. *Comptes Rendus Chim.* **2017**, *20* (3), 283–295. https://doi.org/10.1016/j.crci.2015.11.026.

(8) Droghetti, F.; Lemken, F.; Rulíšek, L.; Ruggi, A.; Natali, M. Selective and Efficient Light-Driven CO2 Reduction to CO with a Heptacoordinated Polypyridine Iron(II) Catalyst. *ACS Catal.* **2024**, *14* (22), 16920–16935. https://doi.org/10.1021/acscatal.4c04290.

(9) Pitre, S. P.; McTiernan, C. D.; Vine, W.; DiPucchio, R.; Grenier, M.; Scaiano, J. C. Visible-Light Actinometry and Intermittent Illumination as Convenient Tools to Study Ru(Bpy)3Cl2 Mediated Photoredox Transformations. *Sci. Rep.* **2015**, *5* (1), 16397. https://doi.org/10.1038/srep16397.

(10) Kinnaird, M. G.; Whitten, D. G. Luminescent Bimetallic Complexes. Study of the Formation, Redox Behavior, and Photochemistry of Complexes between Ag+ and Bis(2,2′-Bipyridine)-Bis-Cyanoruthenium(II). *Chem. Phys. Lett.* **1982**, *88* (3), 275–280. https://doi.org/10.1016/0009-2614(82)87087-5.
